# Supplementary material for: Economic costs of terminal care for selected non-communicable diseases from a healthcare perspective: a review of mortality records from a tertiary hospital in Nigeria
Source: BMJ Open. 2021 Apr 24;11(4):e044969. doi: 10.1136/bmjopen-2020-044969 (PMC8076932; doi:10.1136/bmjopen-2020-044969)
Supplement: Supplementary data [file bmjopen-2020-044969supp001.pdf]

Appendix 1: Average costs (2020 prices) per patient for a month of terminal care for patients with lung cancer, liver cancer and liver cirrhosis

	Admission history (average)			Average costs for patients										
	Average times of admission	Average hospital visits made	Average days on Admission *	Registration	Consultation	Service Charge	Drugs*	Consumables	Admission	Investigations	Bed side Procedures**	Average Direct Medical	Average Direct Non-medical (transport)	Average Direct medical and non-medical
**Lung Ca (n = 5)**														
2020 naira	1.2	1.6	26.7	1959.83	4442.28	1306.55	13128.24	17798.16	39486.94	354061.35	52262.12	484445.46	25707.16[a]	510152.62
2020 dollar				5.44	12.32	3.62	36.42	49.37	109.53	982.14	144.97	1343.82	71.31	1415.13
**Liver Ca (n = 9)**														
2020 naira	1	1	16.6	1959.83	2467.93	725.86	14404.02	36853.50	24840.48	193381.46	26131.06	300764.15	8186.12[b]	308950.27
2020 dollar				5.44	6.85	2.01	39.96	102.23	68.91	536.43	72.49	834.30	22.71	857.00
**Liver cirrhosis (n = 8)**														
2020 naira	1	1	13.9	1959.83	3084.92	907.33	24231.84	18282.67	21231.49	139302.14	26131.06	235131.27	2990.55[c]	238121.83
2020 dollar				5.44	8.56	2.52	67.22	50.71	58.89	386.41	72.49	652.24	8.30	660.53

* Average number of days in total spent in hospital across all of a patient's admissions

a= 4 patients came from other states b = 3 patients came from another state, c =1 patient came from another state

*Drugs listed were mainly supportive for most of the patients

**Examples of bedside procedures - CTTD: Closed tube thoracostomy drainage
