# Supplementary material for: Economic costs of terminal care for selected non-communicable diseases from a healthcare perspective: a review of mortality records from a tertiary hospital in Nigeria
Source: BMJ Open. 2021 Apr 24;11(4):e044969. doi: 10.1136/bmjopen-2020-044969 (PMC8076932; doi:10.1136/bmjopen-2020-044969)
Supplement: Author's manuscript [file bmjopen-2020-044969.draft_revisions.pdf]

BMJ Open is committed to open peer review. As part of this commitment we make the peer review history of every article we publish publicly available.

When an article is published we post the peer reviewers' comments and the authors' responses online. We also post the versions of the paper that were used during peer review. These are the versions that the peer review comments apply to.

The versions of the paper that follow are the versions that were submitted during the peer review process. They are not the versions of record or the final published versions. They should not be cited or distributed as the published version of this manuscript.

BMJ Open is an open access journal and the full, final, typeset and author-corrected version of record of the manuscript is available on our site with no access controls, subscription charges or pay-per-view fees (http://bmjopen.bmj.com).

If you have any questions on BMJ Open's open peer review process please email info.bmjopen@bmj.com

BMJ Open

Economic costs of terminal care for selected non-communicable diseases in Nigeria: A health care perspective

Journal:	*BMJ Open*
Manuscript ID	bmjopen-2020-044969
Article Type:	Original research
Date Submitted by the Author:	23-Sep-2020
Complete List of Authors:	Olumide, Adesola; University of Ibadan College of Medicine, Institute of Child Health<br>Shmueli, Amir; Hebrew University of Jerusalem, School of Public Health,<br>Omotade, Olayemi; University of Ibadan, institute of Child Health<br>Adebayo, Emmanuel; University of Ibadan College of Medicine, Institute of Child Health<br>Alonge, Temitope; University of Ibadan College of Medicine, Surgery<br>Ogun, Gabriel; University of Ibadan College of Medicine, Pathology
Keywords:	Health economics < HEALTH SERVICES ADMINISTRATION & MANAGEMENT, PUBLIC HEALTH, PREVENTIVE MEDICINE, Health policy < HEALTH SERVICES ADMINISTRATION & MANAGEMENT

SCHOLARONE™
Manuscripts

BMJ

I, the Submitting Author has the right to grant and does grant on behalf of all authors of the Work (as defined in the below author licence), an exclusive licence and/or a non-exclusive licence for contributions from authors who are: i) UK Crown employees; ii) where BMJ has agreed a CC-BY licence shall apply, and/or iii) in accordance with the terms applicable for US Federal Government officers or employees acting as part of their official duties; on a worldwide, perpetual, irrevocable, royalty-free basis to BMJ Publishing Group Ltd ("BMJ") its licensees and where the relevant Journal is co-owned by BMJ to the co-owners of the Journal, to publish the Work in this journal and any other BMJ products and to exploit all rights, as set out in our licence.

The Submitting Author accepts and understands that any supply made under these terms is made by BMJ to the Submitting Author unless you are acting as an employee on behalf of your employer or a postgraduate student of an affiliated institution which is paying any applicable article publishing charge ("APC") for Open Access articles. Where the Submitting Author wishes to make the Work available on an Open Access basis (and intends to pay the relevant APC), the terms of reuse of such Open Access shall be governed by a Creative Commons licence – details of these licences and which Creative Commons licence will apply to this Work are set out in our licence referred to above.

Other than as permitted in any relevant BMJ Author's Self Archiving Policies, I confirm this Work has not been accepted for publication elsewhere, is not being considered for publication elsewhere and does not duplicate material already published. I confirm all authors consent to publication of this Work and authorise the granting of this licence.

For peer review only

Economic costs of terminal care for selected non-communicable diseases in Nigeria: A health care perspective

* Adesola O Olumide.[1], Amir Shmueli [2], Olayemi O. Omotade[1], Emmanuel S Adebayo [3], Temitope O. Alonge[4], Gabriel O. Ogun [5]

1. Institute of Child Health, College of Medicine, University of Ibadan and University College Hospital, Ibadan, Nigeria
2. School of Public Health, Hebrew University of Jerusalem, Israel
3. Institute of Child Health, College of Medicine, University of Ibadan Nigeria
4. Department of Surgery, College of Medicine, University of Ibadan and University College Hospital, Ibadan, Nigeria
5. Department of Pathology, College of Medicine, University of Ibadan and University College Hospital, Ibadan, Nigeria

*Corresponding author: Adesola O. Olumide, Institute of Child Health, College of Medicine, University of Ibadan and University College Hospital, Ibadan, Nigeria

Email: daisyolu@yahoo.co.uk; ; aoolumide@com.ui.edu.ng

Short title: Economic costs of NCDs in Nigeria

Key words: Economic costs; Non-communicable disease; Medical costs;

For peer review only - http://bmjopen.bmj.com/site/about/guidelines.xhtml

Economic costs of terminal care for selected non-communicable diseases in Nigeria: A health care perspective

Abstract

Introduction: Data from the World Health Organization revealed that morbidity and mortality from NCDs were on the increase and that NCDs were estimated to account for 29% of all deaths in Nigeria in 2016. This study was conducted to estimate the economic cost of selected non-communicable diseases – lung cancer, liver cancer and liver cirrhosis. These diseases are known to be associated with key modifiable health-risk behaviours (smoking and alcohol use) which are prevalent in Nigeria and often commence during the adolescent years.

Methods: Data were obtained between 2016 and 2017, from mortality records of patients managed for each of the diseases in the University College Hospital, a major referral center in Nigeria. Information on costs of treatment, clinic visits, admission etc. and transportation costs were obtained. Average costs (in 2020 prices) were computed per patient for a month of terminal in-hospital care. Costs were converted to the US dollar equivalent using the current official rate of 1United States Dollar (USD): Nigerian Naira (NGN) 360.50.

Results: All the clients for whom data was obtained were diagnosed in the terminal stages of the disease. The average direct costs were NGN509, 068.40 (USD 1412.12), NGN 529,114.84 (USD 1467.72) and NGN 303,179.08 (USD 840.99) for one month of terminal hospital care for patients managed for lung cancer, liver cancer and liver cirrhosis respectively.

Conclusion: The economic costs of each of the diseases were very high. Findings emphasize the need for aggressive efforts to promote primary prevention in view of the immense economic burden of managing these diseases. Efforts to improve early diagnosis and provide affordable treatment are also necessary in view of the fact that the monthly minimum wage is less than USD 85.00 and treatment costs are borne out-of-pocket by the generality of the population in Nigeria.

For peer review only - http://bmjopen.bmj.com/site/about/guidelines.xhtml

Article summary

Strengths and limitations of this study

- This is a novel study providing direct costs of terminal care for selected Non-Communicable Diseases (NCDs) in Nigeria from a healthcare perspective
- The data collection methods utilized were robust; lending credence to the strength of the findings
- Data were retrieved from a federal government tertiary facility which is a major referral centre for management of Non Communicable Diseases. Treatment costs could therefore differ from costs in private facilities within the country.
- Costs were computed based on information in available patient records, however not all patient records could be retrieved. Costs however still represent a reasonable estimate of terminal costs of care for the selected NCDs.

What is already known?

- The burden of Non-communicable diseases is on the increase in low and middle income countries
- The costs of managing these diseases are significant
- Information on cost of care for Non-Communicable diseases from low and middle income countries is sparse

What are the new findings?

- The costs of terminal care for non-communicable diseases in Nigeria are substantial
- These costs are potentially catastrophic when viewed against the prevailing socio-economic conditions in the country

What do the new findings imply?

- There is an urgent need to implement interventions for preventing NCDs in Nigeria and other LMICs in view of the high economic costs of managing these diseases
- Cost of illness studies should be encouraged in LMICs as the findings can contribute to advocacy efforts to increase expenditure on prevention of these diseases

Keywords

Economic costs; Noncommunicable disease; Medical costs;

For peer review only - http://bmjopen.bmj.com/site/about/guidelines.xhtml

Introduction

Globally, there has been an increase in the prevalence of non-communicable diseases (1). These diseases were previously viewed as diseases of the affluent with a high prevalence in developed countries (2). This is no longer the case however and the larger burden of these diseases is now borne by low and middle income countries(1). While still grappling with the burden from communicable diseases like malaria and pneumonia, developing countries are experiencing an increase in the occurrence of chronic non-communicable diseases (1, 3). Thus, these countries are currently experiencing a double burden of diseases from communicable and non-communicable diseases (1, 3). In Nigeria the disease burden from communicable diseases is still very high, however, the burden from Non-Communicable diseases is on the increase. The World Health Organization estimated that morbidity and mortality from NCDs accounted for approximately 29% of all deaths in the country in 2016 (4). Research has proven that many of the chronic conditions that individuals suffer in adulthood are linked to unhealthy habits e.g. substance including tobacco and alcohol use and unhealthy eating habits and unsafe sexual intercourse that they adopted during their younger years (5-7). Common established outcomes of these health-risk behaviours include the occurrence of various cancers and other non-communicable conditions such as cardiovascular diseases and diabetes later in life (1). These diseases result in considerable morbidity, mortality and economic losses (8) emphasizing the need for continued research to inform policy and practice geared towards their control.

Cost of illness studies determine the burden of diseases to society in monetary terms. In addition, they provide an estimate of the maximum amount that could potentially be saved if a disease were to be eradicated (9-11). Typically, cost of illness studies do not evaluate interventions that address the disease under study. Thus they do not provide information on the benefits of implementing specific interventions or which of a range of interventions would produce maximal impact (11). Cost of illness studies as the name implies, document a range of illness costs which are usually categorized as direct, indirect or intangible costs. Direct costs measure, “the opportunity cost of resources used for treating a particular

For peer review only - http://bmjopen.bmj.com/site/about/guidelines.xhtml

illness" or, "costs for which payments are actually made". Examples of direct costs could be direct medical costs such as costs of treatment, drugs, in-patient admission, etc. or direct non-medical costs such as transportation costs, costs of special meals, relocation costs etc. (11, 12). Indirect costs refer to, "the value of resources lost due to a particular illness", and intangible costs include costs of pain and suffering (11, 12).

There has been an increase in the numbers of studies documenting the cost of various non-communicable diseases, however, the overwhelming majority of these studies have been conducted in developed countries or in developing countries in Asia (13-19). Data from existing studies confirm the immense economic burden from Non-Communicable diseases. Muka et al (2015) in their review of the global impact of non-communicable diseases on healthcare found that globally, cancer and cardiovascular diseases (CVD) had the highest reported mean annual total direct costs, with estimated costs of approximately 197,772 USD and 81,096 USD, respectively (20) A systematic review of cost of diabetes mellitus in Africa by Mutyambizi et al (2018) reported national direct costs of diabetes ranging from 3.5 to 4.5 billion International dollars per annum (21). There remains a paucity of these studies in Sub-Saharan African and in Nigeria, resulting in a reliance on cost estimates from developed countries or developing countries outside of sub-Saharan Africa for drawing inferences for the economic burden of diseases in our clime. The current study was conducted to determine the cost of selected chronic illnesses, namely, lung cancer, liver cancer and chronic liver disease in Ibadan Nigeria. These illnesses were selected because they are recognized long term consequences of some modifiable health-risk behaviours associated with a significant proportion of NCDs, namely smoking and alcohol consumption, they result in significant morbidity and mortality and there are proven measures for primary prevention of the health-risk behaviours they are associated with. Data were obtained as part of a larger study on the predictors and economic costs of selected health-risk behaviours among adolescents in Oyo state, Nigeria.

For peer review only - http://bmjopen.bmj.com/site/about/guidelines.xhtml

Methods

This prevalence-based study adopted a health care system perspective and thus focused on the medical costs of the illnesses. The estimation of the cost of care for the long-term illnesses in this study was guided by the cost-of-illness approach described by Rice (22) and the WHO economic framework of cost estimation described in the World Health Organization Guide to identifying the economic consequences of disease and injury(13, 23)

Data on the illness costs were collected between September 2016 and January, 2017 from the University College Hospital (UCH), Ibadan. Established in 1952, it is the premier teaching hospital in Nigeria and is located in Ibadan, South west Nigeria. The hospital also houses the first cancer registry in Nigeria - the Ibadan Cancer Registry which was established in 1960 within the Pathology department of the University of Ibadan (24). The UCH is a training centre for doctors, nurses and various other categories of health staff at undergraduate and postgraduate levels and also provides tertiary level health services. It is a major receiving centre for tertiary level management of patients with various communicable and non-communicable diseases who are referred from various parts of the country.

We obtained data retrospectively from a review of case records of patients who had been diagnosed and received treatment for the illnesses investigated. The Case note numbers of patients who died from the diseases of interest – lung cancer and liver disease (liver cancer and liver cirrhosis) in 2014 and 2015 were obtained from the Medical records department. There was a total of 12 reported deaths from lung cancer, of these, 5 case notes could be retrieved. In all, 78 patients were reported to have died from liver diseases (liver cirrhosis, liver cancer and chronic liver disease) in 2014 and 2015. Of these, nine case notes of patients with liver cancer and eight for patients with liver cirrhosis could be retrieved. Nine casefiles of patients with chronic liver disease were retrieved but information from these cases notes were not utilized because there was no definitive diagnosis in the case note (this could have occurred because payment was yet to be made for the investigations, hence they were carried out). Relevant information – age, sex, occupation, home address, date of diagnosis, medical treatment, investigations, number of clinic visits, admission

For peer review only

history (number of admissions and duration on admission) etc. were extracted manually from the patient case notes onto a datasheet.

Direct medical (healthcare) costs obtained included the following: cost of hospital admission, clinic visits, investigations, medical procedures, medications, and consumables. The average time from diagnosis to demise for the patients whose case records were obtained ranged from 0 to 3 months indicating that most patients were admitted at the point when the illnesses were at the terminal stages. Costs were obtained from the relevant departments in the hospital. In addition, costs of investigations and drugs were also obtained from a registered private laboratory and a pharmacy within Ibadan. This was because anecdotal reports indicated that some patients procure their drugs or get some of their investigations done outside the hospital. The costs of drug and investigations from these private sources were compared with hospital costs, summed and the average for each item was documented. For each illness investigated (lung cancer, liver cancer, and liver cirrhosis), the total medical costs for each patient was summed and the average costs incurred from the time of diagnosis to demise was estimated for each disease based on information obtained from the patients' case note.

The direct non-medical cost of illness was limited to transportation costs only. Transportation costs were computed for each patient using the home address provided in the case note. A list of all the addresses was compiled and information on transportation costs from these neighbourhoods to the hospital was obtained from informal interviews with commercial transport drivers in the relevant motor-parks. The cost of regular public transportation and the cost of car hire services were obtained. For public transportation, the costs of a return trip for two people was calculated as it was assumed that at least one person would accompany the patient to hospital at every visit because information obtained from the case notes indicated that the patients presented quite late in the course of the disease. For car hire, the cost of hiring the vehicle for a return trip was computed. The average costs (public transportation and car hire) were computed for each patient and

For peer review only - http://bmjopen.bmj.com/site/about/guidelines.xhtml

the average transportation costs per patient calculated for each disease. (See Appendix 1 for table showing average costs per item for each of the three diseases)

For the purposes of this paper, all costs are reported using 2020 as the base year. The 2017 prices were converted to their 2020 values using the average annual inflation rate reported by the Central Bank of Nigeria (CBN) between 2017 and May, 2020 which was 13.23% (25). The costs were then converted to their dollar equivalent using the official CBN June 2020 exchange rate of NGN 360.50: USD1.00.(26) to aid comparison with findings from other studies.

Costs were computed using the formulae below:

(i) Total Direct Cost of Care (DC) for all patients for duration of treatment in days

= Direct medical (DM) + Direct non-medical costs (DN-M)

(ii) Average Direct Cost for all the patients for the duration of treatment in days (ADC)

= DC/number of patients (n)

(iii) Average Direct Cost for all the patients for one month (ADC per patient per month) = Cost of care per patient per month (Cost of Care)

= ADC * (30/average days on treatment)

(See Table 1: Summary of variables used for determining direct medical and non-medical costs)

Ethics Considerations

Ethical approval was obtained from the Oyo State Ethics Committee. Permission to retrieve and review patients' mortality records and case notes was obtained from the University College Hospital management and the heads of the relevant departments. The department heads were assured that information which could identify any patient would not be taken.

For peer review only - http://bmjopen.bmj.com/site/about/guidelines.xhtml

Patient and Public Involvement statement

Patients or the public WERE NOT involved in the design, or conduct, or reporting, or dissemination plans of our research

Results

The mean age of the patients with a diagnosis of lung cancer was, 61 years, male: female ration 3:2. Patients with liver cancer had an average age of 51.1 years with a male: female ratio of 8:1 and patients with liver cirrhosis had a mean age of 51.6 years and all were male.

Medical costs of lung cancer

Records retrieved revealed that on the average, the patients were managed for 0.92 months (from diagnosis to demise) and were on admission for 27.4 days. They made an average of 1.6 trips to the hospital. The average direct medical costs of managing one patient for 0.92 months in 2020 was NGN 484,445. 46 (USD 1,343.82) and transportation cost was NGN 25,707.16 (USD 71.31). Average direct cost of managing one patient for one month was more than 554 thousand naira (USD 1538.18); (Table 2).

Medical cost of liver cancer

Details of the average direct costs for managing a patient with liver cancer for 0.54 months is presented in Table 3. Information was obtained for nine patients who received terminal medical care for an average of 0.54 months (approximately 21 days) from diagnosis to demise. Average direct medical cost was NGN 300764.15 (USD834.30) per patient for a duration of 0.54 months and average transportation cost was NGN 8186.12 (USD22.71). The average direct cost of managing one patient with liver cancer for one month in 2020 was estimated to be NGN 572130.10 (USD1587.05).

Medical costs of liver cirrhosis

Details of the average direct costs for managing a patient with liver cirrhosis in 2020 prices is presented in Table 4. The eight patients for whom information was obtained were managed for an average of 0.71

For peer review only - http://bmjopen.bmj.com/site/about/guidelines.xhtml

months (approximately 21 days). Average direct medical cost was NGN 235131.27 (USD 652.24) per patient for a duration of 0.71 months Average transportation cost was NGN 2990.55 (USD 8.30). All the patients were residing in the same town as the hospital at the time of presentation. Total direct cost of managing one patient with liver cirrhosis for one month of terminal care was estimated to be NGN 335,382.90 (USD 930.33).

Discussion

All the patients for whom data were available presented in the late stages of disease, hence duration of time between diagnosis and demise was very short and patients were on admission for less than a month in virtually all instances. This would have resulted in a reduction in healthcare costs from hospital admission and transportation for follow-up clinic visits documented. Existing literature has highlighted that access and utilization of health care services is still sub-optimal in many developing countries and patients often present late leading to poor disease prognosis (27-29).

Many reasons have been given for delayed presentation of patients in hospital. Patient-related reasons for delayed presentation include inadequate knowledge of the disease and of where to obtain appropriate services, preference for traditional providers over orthodox health facilities, poverty, inadequate transportation (28, 30, 31). On the health facility end, experience of the health worker could affect referral for definitive care (30, 31).

The average direct costs (medical and non-medical) for a month of terminal care for each of the diseases studied was very high. We estimated an average direct cost of approximately five hundred and fifty five thousand naira (USD 1538.18) for managing a patient with lung cancer for one month of terminal care. Other studies reported similar significant medical costs of care for patients with lung cancer. For example, Enstone et al (2018) in their systematic review of the economic burden of small cell lung cancer reported very high direct medical costs (15). Fan et al (2018) reported an average annual direct cost of 63,675 Renminbi (RMB); (USD 10,221, one USD = 6.23 RMB in 2015) per lung cancer patient in China.

For peer review only - http://bmjopen.bmj.com/site/about/guidelines.xhtml

Patients in their study had been diagnosed up to 2 years prior to the study compared with less than a month in our study. Cipriano et al (2011) reported that monthly costs in the first 6 months of care for a 72-year old diagnosed with lung cancer in the United States of America in the year 2000, ranged from USD 2,687 (no active treatment) to USD 9,360 (chemo-radiotherapy) in 2006 prices, although these costs varied by stage at diagnosis and histologic type.(17). Sheenan et al (2019) in a more recent study reported an estimate of USD21,603 for a month of care in the terminal phase, for a patient who died of lung cancer at age 70 in 2017 in the United States (16).

In the current study, the average direct cost of managing one patient with liver cancer in Ibadan, Nigeria for one month in 2020 was estimated to be NGN 572,130.10 (USD1587.05). Gondek et al (2008) estimated that the annual cost of HCC in the US was approximately $31,641 per patient (14). The total direct cost of managing one patient with liver cirrhosis for one month of terminal care in our study was estimated to be NGN 335, 382.90 (USD 930.33). In a study of patients with cirrhosis in Vietnem, Nguyen et al (2017) estimated that the median cost of treatment per session in 2015 was 7,439,527.40 ± 5,251,403.97 VND; i.e. approximately USD 329.90±232.88 (1 US Dollar in Vietnamese Dong: USD22,550.18 for 12/31/2015) (32) of which almost 69% of the costs were covered by health insurance (18).

Although comparisons between our estimates and those provided by the studies cited above need to be made with caution because of variations in the disease stage, exact types and duration of treatment provided (among other variables), our findings show that irrespective of the setting, the amount expended to treat each of the diseases considered is significant. Boutayeb and Boutayeb (2005) in their review of the burden of non-communicable diseases in developing countries highlighted the huge difference in costs expended in managing NCDs between patients in developed and developing countries and between affluent and poor patients within developed countries noting that this reflected inequity in access to treatment. In developed countries, a significant proportion of the treatment costs are covered by health insurance and availability of free or largely subsidized care promotes early presentation of patients. This is unlike the situation in Nigeria and many lower income countries where a significant proportion of the population lives on less than a dollar

For peer review only - http://bmjopen.bmj.com/site/about/guidelines.xhtml

a day and treatment costs are largely borne out-of-pocket. Thus these costs are catastrophic for many families and further explain why many patients present late or default after initial presentation and thus their data does not get captured in formal mortality data.

Conclusion

Our study has demonstrated that direct medical costs of lung and liver cancer and liver cirrhosis in Ibadan, Nigeria are very high, especially when viewed against the prevailing economic environment of the country. Furthermore, many patients in this setting present late resulting in poor prognosis. The substantial costs of care further emphasizes the need for greater efforts at prevention of these diseases. Major risk factors for each of the diseases investigated have been identified and interventions exist to reduce engagement in these risky practices. Furthermore, early diagnosis which can only occur if patients present early and are diagnosed on time needs to be urgently addressed. This would improve disease outcome in the patients and possible reduce medical costs.

Limitations

1. The hospital data used to compute the average costs of long-term illness were obtained from a patients' records in their case notes. However, all the case notes could not be retrieved. This was because patient records are yet to be fully computerized, hence the case notes had to be manually retrieved. However, all case notes were not found because some were yet to be returned to the central records office as they were still being processed administratively. In addition, records found were for patients who had an average survival of less than a month indicating late stage disease. This could have resulted in an under-estimation of the overall costs incurred. The costs presented are described as costs of a month of terminal care.
2. Transportation costs were based on the initial addresses documented in the patients' case notes and it was not possible to determine if the patients had re-located closer to the hospital during the course of the illness. This could have led to an over-estimation of the costs for the patients who relocated

For peer review only - http://bmjopen.bmj.com/site/about/guidelines.xhtml

closer to the hospital although costs of relocating to a closer location could offset this difference. The costs however still provide a reasonable estimation of the transportation costs.

3. The hospital is a federal government tertiary facility, however, treatment costs could therefore differ from costs in other federal and private facilities within the country.

Inspite of these limitations, our findings are still very useful and represent estimates of costs of terminal care for lung cancer and liver cancer and cirrhosis in our setting.

Funding statement

This research was supported by the Consortium for Advanced Research Training in Africa (CARTA). CARTA is jointly led by the African Population and Health Research Center and the University of the Witwatersrand and funded by the Carnegie Corporation of New York (Grant No. G-16-54067), Sida (Grant No:54100113), the DELTAS Africa Initiative (Grant No: 107768/Z/15/Z). The DELTAS Africa Initiative is an independent funding scheme of the African Academy of Sciences (AAS)'s Alliance for Accelerating Excellence in Science in Africa (AESA) and supported by the New Partnership for Africa's Development Planning and Coordinating Agency (NEPAD Agency) with funding from the Wellcome Trust (UK) and the UK government. The statements made and views expressed are solely the responsibility of the Fellow.

Acknowledgements

All research staff and respondents, management and heads of relevant departments of the University College Hospital, Ibadan are appreciated.

Competing interests

The authors declare that they have no known competing interests

Authors' contributions

AO conceptualized and developed the protocol for the main study from which the dataset for the current paper was obtained as this formed the research for her doctoral thesis. AO conducted the literature search,

For peer review only - http://bmjopen.bmj.com/site/about/guidelines.xhtml
For peer review only

and was involved in data collection, analysis and write-up of the current paper. AS and OO were AO's doctoral supervisors. They supervised the entire project and were involved in the write up and revision of the current paper. EA was involved in data collection, analysis and write-up of the data for the current paper. TA and GO collated the clinical information of patients who died in the hospital and generated a list of all-cause mortality for patients managed in the teaching hospital. All authors read and approved the final manuscript.

Data sharing statement

No additional data available

References

1. Habib SH, Saha S. Burden of non-communicable disease: Global overview. Diabetes & Metabolic Syndrome: Clinical Research & Reviews. 2010 2010/01/01/;4(1):41-7.
2. World Health Organization. Rethinking "diseases of affluence"the economic impact of chronic diseases. WHO, 2005.
3. Boutayeb A, Boutayeb S. The burden of non communicable diseases in developing countries. International journal for equity in health. 2005 Jan 14;4(1):2. PubMed PMID: 15651987. Pubmed Central PMCID: PMC546417. Epub 2005/01/18. eng.
4. World Health Organization. Noncommunicable Diseases (NCD) Country Profiles2018. Available from: https://www.who.int/nmh/countries/nga_en.pdf?ua=1.
5. World Health Organization. Health for the world's adolescents: a second chance in the second decade: summary. World Health Organization, 2014.
6. Alwan A, Maclean DR, Riley L, et al. Monitoring and surveillance of chronic non-communicable diseases: progress and capacity in high-burden countries. Lancet. 2010;376:1861 - 68.
7. Beaglehole R, Bonita R, Horton R, Adams C, Alleyne G, Asaria P, et al. Priority actions for the non-communicable disease crisis. Lancet. 2011 Apr 23;377(9775):1438-47. PubMed PMID: 21474174. Epub 2011/04/09. eng.
8. Jaspers L, Colpani V, Chaker L, van der Lee SJ, Muka T, Imo D, et al. The global impact of non-communicable diseases on households and impoverishment: a systematic review. European Journal of Epidemiology. 2015;30(3):163-88.
9. Rice D. Cost-of-illness studies: fact or fiction? The Lancet. 1994;344(8936):1519-20.

For peer review only - http://bmjopen.bmj.com/site/about/guidelines.xhtml

10. Rice DP. Cost of illness studies: what is good about them? Injury Prevention. 2000;6(3):177-9.

11. Segel JE. Cost-of-illness studies—a primer. RTI-UNC Center of Excellence in Health Promotion Economics. 2006:1-39.

12. Rice DP, Kelman S, Miller LS. Estimates of economic costs of alcohol and drug abuse and mental illness, 1985 and 1988. Public health reports. 1991;106(3):280.

13. World Health Organization. WHO guide to identifying the economic consequences of disease and injury. 2009.

14. Gondek K, Lang K, Danchenko N, Shah S, Anderson S, Thompson D. Economic costs of hepatocellular carcinoma in the United States. Journal of Clinical Oncology. 2008;26(15_suppl):6555-.

15. Enstone A, Greaney M, Povsic M, Wyn R, Penrod JR, Yuan Y. The economic burden of small cell lung cancer: a systematic review of the literature. PharmacoEconomics-open. 2018;2(2):125-39.

16. Sheehan DF, Criss SD, Chen Y, Eckel A, Palazzo L, Tramontano AC, et al. Lung cancer costs by treatment strategy and phase of care among patients enrolled in Medicare. Cancer medicine. 2019 Jan;8(1):94-103. PubMed PMID: 30575329. Pubmed Central PMCID: PMC6346221. Epub 2018/12/24. eng.

17. Cipriano LE, Romanus D, Earle CC, Neville BA, Halpern EF, Gazelle GS, et al. Lung cancer treatment costs, including patient responsibility, by disease stage and treatment modality, 1992 to 2003. Value Health. 2011;14(1):41-52. PubMed PMID: 21211485. eng.

18. Nguyen D, Nguyen T, Pho N. Analyzing the treatment cost of liver cirrhosis from healthcare payer's perspective of Vietnam in 2015. Value in Health. 2017;20(9):A633.

19. Rezaei S, Akbari Sari A, Arab M, Majdzadeh R, Mohammad Poorasl A. Economic burden of smoking: a systematic review of direct and indirect costs. Med J Islam Repub Iran. 2016;30:397-. PubMed PMID: 27579287. eng.

20. Muka T, Imo D, Jaspers L, Colpani V, Chaker L, van der Lee SJ, et al. The global impact of non-communicable diseases on healthcare spending and national income: a systematic review. European journal of epidemiology. 2015;30(4):251-77.

21. Mutyambizi C, Pavlova M, Chola L, Hongoro C, Groot W. Cost of diabetes mellitus in Africa: a systematic review of existing literature. Globalization and Health. 2018 2018/01/16;14(1):3.

22. Rice D. Estimating the cost of illness: health economics series No. 6, PHS No. 947–6. Washington, DC: US Government Printing Office. 1966.

23. Chisholm D, Stanciole AE, Tan Torres Edejer T, Evans DB. Economic impact of disease and injury: counting what matters. BMJ. 2010;340.

For peer review only

For peer review only - http://bmjopen.bmj.com/site/about/guidelines.xhtml

24. Jedy-Agba E, Curado MP, Ogunbiyi O, Oga E, Fabowale T, Igbinoba F, et al. Cancer Incidence in Nigeria: A Report from Population-based Cancer Registries. Cancer epidemiology. 2012 05/22;36(5):e271-e8. PubMed PMID: PMC3438369.

25. CBN. Central Bank of Nigeria: Data and Statistics - Inflation rates n.d. [cited 2017 26 July]. Available from: https://www.cbn.gov.ng/rates/inflrates.asp.

26. CBN. Central Bank of Nigeria: Exchange rate archives: Central Bank of Nigeria; n. d. [cited 2017 6 March, 2017]. Available from: http://www.cbn.gov.ng/rates/ExchangeArchives.asp.

27. Igwegbe AO, Eleje GU, Ugboaja JO, Ofiaeli RO. Improving maternal mortality at a university teaching hospital in Nnewi, Nigeria. International Journal of Gynecology & Obstetrics. 2012 2012/03/01/;116(3):197-200.

28. Ezeome E. Delays in presentation and treatment of breast cancer in Enugu, Nigeria. Nigerian journal of clinical practice. 2010;13(3).

29. Akande TM, Owayemi J. Health-seeking behaviour in Anyigba, North-Central Nigeria. Res J Med Sci. 2009;3:47-51.

30. Almuammar A, Dryden C, Burr J. Factors associated with late presentation of cancer: a limited literature review. Journal of Radiotherapy in Practice. 2010;9(2):117.

31. Idowu OE, Apemiye RA. Delay in presentation and diagnosis of adult primary intracranial neoplasms in a tropical teaching hospital: A pilot study. International Journal of Surgery. 2009 2009/01/01/;7(4):396-8.

32. rates.org. E. [20 June, 2020]. Available from: https://www.exchange-rates.org/Rate/USD/VND/12-31-2015.

For peer review only

For peer review only - http://bmjopen.bmj.com/site/about/guidelines.xhtml

Table 1: Summary of variables used for determining direct medical and non-medical costs

DM for all patients with the illness	DN-M for all patients with the illness	DC for all patients with the illness	Average DC per patients in days (ADC)	ADC per patient per month (Cost of care)
DM	DN-M	DM+DN-M	DC/ n	ADC*(30/days on treatment)

For peer review only

For peer review only - http://bmjopen.bmj.com/site/about/guidelines.xhtml

Table 2: Average direct medical and transportation costs for managing patients who died from lung cancer in 2020

Duration of treatment* (months)	Average no. of hospital visits	Average No. of days on admission	Average direct medical costs per person	Average direct non-medical costs (transport)	Average direct costs per person for 0.92 months	Average direct costs per person for 1 month
A	B	C	D	E	F = D+E	F/0.92
0.92	1.6	27.4	NGN 484,445.46	NGN 25707.16	NGN 510,152.62	NGN 554,513.70
			USD 1343.82	USD 71.31	USD 1415.13	USD 1538.18

For peer review only

For peer review only - http://bmjopen.bmj.com/site/about/guidelines.xhtml

Table 3 Average direct medical and transportation costs for managing patients who died from liver cancer in 2020

Duration of treatment* (months)	Average no. of hospital visits	Average No. of days on admission	Average direct medical cost per person	Average direct non-medical cost (transport)	Average direct cost per person for 0.54 months	Average direct cost per person for one month
A	B	C	D	E	F = D+E	F/0.54
0.54	1	17.1	NGN 300764.15 USD 834.30	NGN 8,186.12 USD 22.71	NGN 308950.27 USD 857.00	NGN 572130.10 USD1587.05

For peer review only - http://bmjopen.bmj.com/site/about/guidelines.xhtml

Table 4: Average direct medical and transportation costs for managing patients who died from liver cirrhosis in 2020

Duration of treatment* (months)	Average no. of hospital visits	Average No. of days on admission	Average direct medical costs per person	Average direct non-medical costs (transport)	Average direct costs per person for 0.71 months	Average direct costs per person for 1 month
A	B	C	D	E	F = D+E	F/0.71
0.71	1	14.6	NGN 235131.27	NGN 2,990.55	NGN 238121.83	NGN 335382.90
			USD 652.24	USD 8.30	USD 660.53	USD 930.33

For peer review only - http://bmjopen.bmj.com/site/about/guidelines.xhtml

Appendix 1: Average costs (2020 prices) per patient for a month of terminal care for patients with lung cancer, liver cancer and liver cirrhosis

	Admission history (average)			Average costs for patients										
	Average times of admission	Average hospital visits made	Average days on Admission	Registration	Consultation	Service Charge	Drugs*	Consumables	Admission	Investigations	Bed side Procedures (CTTD)	Average Direct Medical	Average Direct Non-medical (transport)	Average Direct medical and non-medical
**Lung Ca (n = 5)**														
2020 naira	1.2	1.6	27.4	1959.83	4442.28	1306.55	13128.24	17798.16	39486.94	354061.35	52262.12	484445.46	25707.16[a]	510152.62
2020 dollar				5.44	12.32	3.62	36.42	49.37	109.53	982.14	144.97	1343.82	71.31	1415.13
**Liver Ca (n = 9)**														
2020 naira	1	1	17.1	1959.83	2467.93	725.86	14404.02	36853.50	24840.48	193381.46	26131.06	300764.15	8186.12[b]	308950.27
2020 dollar				5.44	6.85	2.01	39.96	102.23	68.91	536.43	72.49	834.30	22.71	857.00
**Liver cirrhosis (n = 8)**														
2020 naira	1	1.25	14.6	1959.83	3084.92	907.33	24231.84	18282.67	21231.49	139302.14	26131.06	235131.27	2990.55[c]	238121.82
2020 dollar				5.44	8.56	2.52	67.22	50.71	58.89	386.41	72.49	652.24	8.30	660.53

a= 4 patients came from other states b = 3 patients came from another state, c =1 patient came from another state

*drugs listed were mainly supportive for most of the patients

STROBE Statement—checklist of items that should be included in reports of observational studies

	Item No	Recommendation
**Title and abstract**	1	(*a*) Indicate the study's design with a commonly used term in the title or the abstract
		(*b*) Provide in the abstract an informative and balanced summary of what was done and what was found
		Author comment: Abstract included in line with journal specifications Page 3 of 24
**Introduction**		
Background/rationale	2	Explain the scientific background and rationale for the investigation being reported
		Author comment: Information in Background of paper – Pages 5 and 6 of 24
Objectives	3	State specific objectives, including any prespecified hypotheses
		Author comment: Information is in the concluding paragraph of the introduction, just before the results.- Page 6 of 24
**Methods**		
Study design	4	Present key elements of study design early in the paper
		Author comment: Information in opening paragraph of the methods –Page 7 of 24
Setting	5	Describe the setting, locations, and relevant dates, including periods of recruitment, exposure, follow-up, and data collection
		Author comment: In methods – paragraphs 2 and 3 – Page 7 of 24
Participants	6	(*a*) *Cohort study*—Give the eligibility criteria, and the sources and methods of selection of participants. Describe methods of follow-up<br>*Case-control study*—Give the eligibility criteria, and the sources and methods of case ascertainment and control selection. Give the rationale for the choice of cases and controls<br>*Cross-sectional study*—Give the eligibility criteria, and the sources and methods of selection of participants<br>Author comment: Information in paragraph 3 of methods Page 7 of 24
		(*b*) *Cohort study*—For matched studies, give matching criteria and number of exposed and unexposed<br>*Case-control study*—For matched studies, give matching criteria and the number of controls per case
Variables	7	Clearly define all outcomes, exposures, predictors, potential confounders, and effect modifiers. Give diagnostic criteria, if applicable<br>Author comment: Paragraphs 3, 4 and 5 of the methods section on pages 7 to 8 of 24
Data sources/ measurement	8*	For each variable of interest, give sources of data and details of methods of assessment (measurement). Describe comparability of assessment methods if there is more than one group<br>Author comment Paragraphs 3, 4 and 5 of the methods section on pages 7 to 8 of 24
Bias	9	Describe any efforts to address potential sources of bias<br>Author comment: Medical and transportation costs were obtained from at least two sources. Information presented in Pages 7 and 8 of 24
Study size	10	Explain how the study size was arrived at<br>Author comment: Primary data was not collected and all available records found were analysed.
Quantitative variables	11	Explain how quantitative variables were handled in the analyses. If applicable, describe which groupings were chosen and why

For peer review only

For peer review only - http://bmjopen.bmj.com/site/about/guidelines.xhtml

		Author comment: Information on variables is provided in the section just preceding the ethical approval section Pages 7 to 9 of 24
Statistical methods	12	(*a*) Describe all statistical methods, including those used to control for confounding
		(*b*) Describe any methods used to examine subgroups and interactions
		(*c*) Explain how missing data were addressed
		(*d*) *Cohort study*—If applicable, explain how loss to follow-up was addressed<br>*Case-control study*—If applicable, explain how matching of cases and controls was addressed<br>*Cross-sectional study*—If applicable, describe analytical methods taking account of sampling strategy
		(*e*) Describe any sensitivity analyses
		Author comment: Information presented in the methods section – the sub-section just preceding the results Pages 8 and 9 of 24

Continued on next page

For peer review only

For peer review only - http://bmjopen.bmj.com/site/about/guidelines.xhtml

**Results**		
Participants	13*	(a) Report numbers of individuals at each stage of study—eg numbers potentially eligible, examined for eligibility, confirmed eligible, included in the study, completing follow-up, and analysed - Methods section Page 7 of 24 second paragraph
		(b) Give reasons for non-participation at each stage
		(c) Consider use of a flow diagram
Descriptive data	14*	(a) Give characteristics of study participants (eg demographic, clinical, social) and information on exposures and potential confounders
		(b) Indicate number of participants with missing data for each variable of interest
		(c) *Cohort study*—Summarise follow-up time (eg, average and total amount) Page 10. Opening paragraph of the results section: Page 10 of 24
Outcome data	15*	*Cohort study*—Report numbers of outcome events or summary measures over time
		Case-control study—Report numbers in each exposure category, or summary measures of exposure
		Cross-sectional study—Report numbers of outcome events or summary measures
Main results	16	(*a*) Give unadjusted estimates and, if applicable, confounder-adjusted estimates and their precision (eg, 95% confidence interval). Make clear which confounders were adjusted for and why they were included
		(*b*) Report category boundaries when continuous variables were categorized
		(*c*) If relevant, consider translating estimates of relative risk into absolute risk for a meaningful time period Not applicable
Other analyses	17	Report other analyses done—eg analyses of subgroups and interactions, and sensitivity analyses
		Author comment: Information presented in the results section – Pages 10 to 11 of 24
**Discussion**		**Author comment: Information presented in the results section – Pages 11 to 13 of 24**
Key results	18	Summarise key results with reference to study objectives
Limitations	19	Discuss limitations of the study, taking into account sources of potential bias or imprecision. Discuss both direction and magnitude of any potential bias Author comment: Information presented after the concluding sub-section –Pages 13 and 14 of 24
Interpretation	20	Give a cautious overall interpretation of results considering objectives, limitations, multiplicity of analyses, results from similar studies, and other relevant evidence Author comment: Information provided in the discussion section - Pages 11 to 13 of 24
Generalisability	21	Discuss the generalisability (external validity) of the study results
		Author comment: Information presented in the conclusion section Page 13 of 24
**Other information**		
Funding	22	Give the source of funding and the role of the funders for the present study and, if applicable, for the original study on which the present article is based Author comment: Information presented after the limitations Page 14 of 24

*Give information separately for cases and controls in case-control studies and, if applicable, for exposed and unexposed groups in cohort and cross-sectional studies.

Note: An Explanation and Elaboration article discusses each checklist item and gives methodological background and published examples of transparent reporting. The STROBE checklist is best used in conjunction with this article (freely available on the Web sites of PLoS Medicine at http://www.plosmedicine.org/, Annals of Internal Medicine at

For peer review only

For peer review only - http://bmjopen.bmj.com/site/about/guidelines.xhtml

http://www.annals.org/, and Epidemiology at http://www.epidem.com/). Information on the STROBE Initiative is available at www.strobe-statement.org.

For peer review only

For peer review only - http://bmjopen.bmj.com/site/about/guidelines.xhtml

BMJ Open

Economic costs of terminal care for selected non-communicable diseases from a healthcare perspective: A retrospective review of mortality records from a tertiary hospital in Nigeria

Journal:	*BMJ Open*
Manuscript ID	bmjopen-2020-044969.R1
Article Type:	Original research
Date Submitted by the Author:	11-Feb-2021

Complete List of Authors:	Olumide, Adesola; University of Ibadan College of Medicine, Institute of Child Health<br>Shmueli, Amir; Hebrew University of Jerusalem Braun School of Public Health and Community Medicine, School of Public Health,<br>Omotade, Olayemi; University of Ibadan, College of Medicine, Institute of Child Health<br>Adebayo, Emmanuel; University of Ibadan College of Medicine, Institute of Child Health<br>Alonge, Temitope; University of Ibadan College of Medicine, Surgery<br>Ogun, Gabriel; University of Ibadan College of Medicine, Pathology

**Primary Subject Heading**:	Public health
Secondary Subject Heading:	Health economics
Keywords:	PUBLIC HEALTH, PREVENTIVE MEDICINE, Health policy < HEALTH SERVICES ADMINISTRATION & MANAGEMENT, Health economics < HEALTH SERVICES ADMINISTRATION & MANAGEMENT

SCHOLARONE™
Manuscripts

I, the Submitting Author has the right to grant and does grant on behalf of all authors of the Work (as defined in the below author licence), an exclusive licence and/or a non-exclusive licence for contributions from authors who are: i) UK Crown employees; ii) where BMJ has agreed a CC-BY licence shall apply, and/or iii) in accordance with the terms applicable for US Federal Government officers or employees acting as part of their official duties; on a worldwide, perpetual, irrevocable, royalty-free basis to BMJ Publishing Group Ltd ("BMJ") its licensees and where the relevant Journal is co-owned by BMJ to the co-owners of the Journal, to publish the Work in this journal and any other BMJ products and to exploit all rights, as set out in our licence.

The Submitting Author accepts and understands that any supply made under these terms is made by BMJ to the Submitting Author unless you are acting as an employee on behalf of your employer or a postgraduate student of an affiliated institution which is paying any applicable article publishing charge ("APC") for Open Access articles. Where the Submitting Author wishes to make the Work available on an Open Access basis (and intends to pay the relevant APC), the terms of reuse of such Open Access shall be governed by a Creative Commons licence – details of these licences and which Creative Commons licence will apply to this Work are set out in our licence referred to above.

Other than as permitted in any relevant BMJ Author's Self Archiving Policies, I confirm this Work has not been accepted for publication elsewhere, is not being considered for publication elsewhere and does not duplicate material already published. I confirm all authors consent to publication of this Work and authorise the granting of this licence.

For peer review only

Economic costs of terminal care for selected non-communicable diseases from a healthcare perspective: A retrospective review of mortality records from a tertiary hospital in Nigeria

* Adesola O Olumide.[1], Amir Shmueli [2], Olayemi O. Omotade[1], Emmanuel S Adebayo [3], Temitope O. Alonge[4], Gabriel O. Ogun [5]

1. Institute of Child Health, College of Medicine, University of Ibadan and University College Hospital, Ibadan, Nigeria
2. School of Public Health, Hebrew University of Jerusalem, Israel
3. Institute of Child Health, College of Medicine, University of Ibadan Nigeria
4. Department of Surgery, College of Medicine, University of Ibadan and University College Hospital, Ibadan, Nigeria
5. Department of Pathology, College of Medicine, University of Ibadan and University College Hospital, Ibadan, Nigeria

*Corresponding author: Adesola O. Olumide, Institute of Child Health, College of Medicine, University of Ibadan and University College Hospital, Ibadan, Nigeria

Email: daisyolu@yahoo.co.uk; ; aoolumide@com.ui.edu.ng

Short title: Economic costs of terminal care for selected NCDs in Nigeria

Key words: Economic costs; Non-communicable disease; Lung CA; Liver Ca; Liver cirrhosis; Medical costs;

For peer review only

Economic costs of terminal care for selected non-communicable diseases from a healthcare perspective: A retrospective review of mortality records from a tertiary hospital in Nigeria

Abstract

Introduction: The World Health Organization revealed that morbidity and mortality from Non-Communicable Diseases (NCDs) are on the increase and NCDs accounted for approximately 29% of all deaths in Nigeria in 2016. This study was conducted to estimate the economic cost of selected non-communicable diseases – lung cancer, liver cancer and liver cirrhosis. These diseases are known to be associated with key modifiable health-risk behaviours (smoking and alcohol use) which are prevalent in Nigeria and often commence during the adolescent years.

Methods: Data were obtained between 2016 and 2017, from mortality records of patients managed for the selected diseases in the University College Hospital, a major referral center in Nigeria. Information on costs of treatment, clinic visits, admission etc. and transportation were obtained. Average costs of terminal in-hospital care and transportation costs (in 2020 prices) were computed per patient. Costs were converted to the United States (US) dollar equivalent using the current official rate of USD 1: Nigerian Naira (NGN) 360.50.

Results: Twenty-two (out of 90 cases recorded) could be retrieved and all the patients had been diagnosed in the terminal stages of the disease. The average direct costs were NGN510, 152.62 (USD 1415.13) for an average of 49.2 days of terminal care for lung cancer; NGN 308,950.27 (USD 857.00) and NGN 238,121.83 (USD 660.53) for an average of 16.6 and 21.7 days of terminal care for patients managed for liver cancer and liver cirrhosis respectively.

Conclusion: The economic costs of each of the diseases were very high. Findings emphasize the need for aggressive efforts to promote primary prevention, improve early diagnosis and provide affordable treatment in view of the fact that the monthly minimum wage is less than USD 85.00 and treatment costs are borne out-of-pocket by the generality of the population in Nigeria.

Article summary

For peer review only - http://bmjopen.bmj.com/site/about/guidelines.xhtml

Strengths and limitations of this study

- This is a novel study providing direct costs of terminal care for selected Non-Communicable Diseases (NCDs) in Nigeria from a healthcare perspective
- The data collection methods utilized were robust; lending credence to the strength of the findings
- Costs are based on data retrieved from a Federal government tertiary hospital in Nigeria which are lower than equivalent costs obtainable in private facilities.
- Cost could only be computed for the 22 records retrieved (representing 24.4% of all related mortalities within the time-frame studied)
- In spite of the small sample, the findings are reasonable estimates of direct costs of terminal care for the selected NCDs in the study setting.

Keywords

Economic costs; Noncommunicable disease; Medical costs; Lung cancer, Liver cancer, Liver cirrhosis

Introduction

Globally, there has been an increase in the prevalence of non-communicable diseases (1). These diseases were previously viewed as diseases of the affluent with a high prevalence in developed countries (2). This is no longer the case however and the larger burden of these diseases is now borne by low and middle income countries(1). While still grappling with the burden from communicable diseases like malaria and pneumonia, developing countries are experiencing an increase in the occurrence of chronic non-communicable diseases (1, 3). Thus, these countries are currently experiencing a double burden of diseases from communicable and non-communicable diseases (1, 3). Research has proven that many of the chronic conditions that individuals suffer in adulthood are linked to unhealthy habits e.g. substance including tobacco and alcohol use and unhealthy eating habits and unsafe sexual intercourse that they adopted during their younger years (4-6). Common established outcomes of these health-risk behaviours include the occurrence of various cancers and other non-communicable conditions such as cardiovascular diseases and

For peer review only

diabetes later in life (1). These diseases result in considerable morbidity, mortality and economic losses (7) emphasizing the need for continued research to inform policy and practice geared towards their control.

In Nigeria the disease burden from communicable diseases is still very high, however, available data indicate that the burden from Non-Communicable diseases is significant. The World Health Organization estimated that morbidity and mortality from NCDs accounted for approximately 29% of all deaths in the country in 2016 (8). According to 2020 data from the Global cancer observatory, there were about 124,815 new cases of cancer in Nigeria. Liver and lung cancer were among the top 20 causes of cancer in Nigeria with incidence rates of 4.4% and 1.4% respectively (9). Furthermore of the estimated 78,899 deaths from cancer, 5,180 and 1,789 were from liver and lung cancer respectively. Adekanle, et al (2020) in their review of records of patients admitted with liver diseases into a tertiary hospital in Ile-Ife, Nigeria, from 2013–2017 found that hepatocellular carcinoma and liver cirrhosis accounted for 52.8% and 27.2% of all cases of liver disease admitted during the review period (10).

Cost of illness studies determine the burden of diseases to society in monetary terms. In addition, they provide an estimate of the amount that could potentially be saved if a disease were to be eradicated (11-13). Typically, cost of illness studies do not evaluate interventions that address the disease under study. Thus they do not provide information on the benefits of implementing specific interventions or which of a range of interventions would produce maximal impact (13). Cost of illness studies as the name implies, document a range of illness costs which are usually categorized as direct, indirect or intangible costs. Direct costs measure, “the opportunity cost of resources used for treating a particular illness” or, “costs for which payments are actually made”. Examples of direct costs could be direct medical costs such as costs of treatment, drugs, in-patient admission, etc. or direct non-medical costs such as transportation costs, costs of special meals, relocation costs etc. (13, 14). Indirect costs refer to, “the value of resources lost due to a particular illness”, and intangible costs include costs of pain and suffering (13, 14).

For peer review only - http://bmjopen.bmj.com/site/about/guidelines.xhtml

There has been an increase in the numbers of studies documenting the cost of various non-communicable diseases, however, the overwhelming majority of these studies have been conducted in developed countries or in developing countries in Asia (15-21). Data from existing studies confirm the immense economic burden from Non-Communicable diseases. Muka et al (2015) in their review of the global impact of non-communicable diseases on healthcare found that globally, cancer and cardiovascular diseases (CVD) had the highest reported mean annual total direct costs, with estimated costs of approximately 197,772 USD and 81,096 USD, respectively (22) A systematic review of cost of diabetes mellitus in Africa by Mutyambizi et al (2018) reported national direct costs of diabetes ranging from 3.5 to 4.5 billion International dollars per annum (23). There remains a paucity of these studies in Sub-Saharan African and in Nigeria, resulting in a reliance on cost estimates from developed countries or developing countries outside of sub-Saharan Africa for drawing inferences for the economic burden of diseases in our **region**. The current study was conducted to determine the cost of selected chronic illnesses, namely, lung cancer, liver cancer and chronic liver disease in Ibadan Nigeria. These illnesses were selected because they are recognized long term consequences of some modifiable health-risk behaviours associated with a significant proportion of NCDs, namely smoking and alcohol consumption, they result in significant morbidity and mortality and there are proven measures for primary prevention of the health-risk behaviours they are associated with. Data were obtained as part of a larger study on the predictors and economic costs of selected health-risk behaviours among adolescents in Oyo state, Nigeria.

Methods

This prevalence-based study adopted a health care system perspective and thus focused on the medical costs of the illnesses. The estimation of the cost of care for the long-term illnesses in this study was guided by the cost-of-illness approach described by Rice (24) and the WHO economic framework of cost estimation described in the World Health Organization Guide to identifying the economic consequences of disease and injury (15, 25)

For peer review only - http://bmjopen.bmj.com/site/about/guidelines.xhtml

Data on the illness costs were collected between September 2016 and January, 2017 from the University College Hospital (UCH), Ibadan. Established in 1952, it is the premier teaching hospital in Nigeria and is located in Ibadan, South west Nigeria. The hospital also houses the first cancer registry in Nigeria - the Ibadan Cancer Registry which was established in 1960 within the Pathology department of the University of Ibadan (26). The UCH is a training centre for doctors, nurses and various other categories of health staff at undergraduate and postgraduate levels and also provides tertiary level health services. It is a major receiving centre for tertiary level management of patients with various communicable and non-communicable diseases who are referred from various parts of the country.

We obtained data retrospectively from a review of case records of patients who had been diagnosed and received treatment for the illnesses investigated. The Case note numbers of patients who died from the diseases of interest – lung cancer and liver disease (liver cancer and liver cirrhosis) in 2014 and 2015 were obtained from the Medical records department. There was a total of 12 reported deaths from lung cancer, of these, 5 case notes could be retrieved. In all, 78 patients were reported to have died from liver diseases (liver cirrhosis, liver cancer and chronic liver disease) in 2014 and 2015. Of these, nine case notes of patients with liver cancer and eight for patients with liver cirrhosis could be retrieved. Nine casefiles of patients with chronic liver disease were retrieved but information from these cases notes were not utilized because there was no definitive diagnosis in the case note (this could have occurred because payment was yet to be made for the investigations, hence they were carried out). Ladep et al (2007) had noted that some other challenges to a definitive diagnosis of liver cirrhosis in Nigeria which were reluctance to give consent for liver biopsy and inadequate trained doctors to conduct the procedure (27). Relevant information – age, sex, home address, date of diagnosis, medical treatment, investigations, number of clinic visits, admission history (number of admissions and duration on admission) etc. were extracted manually from the patient case notes onto a datasheet.

Direct medical (healthcare) costs obtained included the following: cost of hospital admission, clinic visits, investigations, medical procedures, medications, and consumables. The average time from presentation

For peer review only - http://bmjopen.bmj.com/site/about/guidelines.xhtml

with symptoms to demise for the patients whose case records were obtained ranged from 0 to about four and a half months indicating that most patients were admitted at the point when the illnesses were at the terminal stages. Costs were obtained from the relevant departments in the hospital. In addition, costs of investigations and drugs were also obtained from a registered private laboratory and a pharmacy within Ibadan. This was because anecdotal reports indicated that some patients procure their drugs or get some of their investigations done outside the hospital. The costs of drug and investigations from these private sources were compared with hospital costs, summed and the average for each item was documented. For each illness investigated (lung cancer, liver cancer, and liver cirrhosis), the total medical costs for each patient was summed and the average costs incurred from the time of diagnosis to demise was estimated for each disease based on information obtained from the patients' case note.

The direct non-medical cost of illness was limited to transportation costs only. Transportation costs were computed for each patient using the home address provided in the case note. A list of all the addresses was compiled and information on transportation costs from these neighbourhoods to the hospital was obtained from informal interviews with commercial transport drivers in the relevant motor-parks. The cost of regular public transportation and the cost of car hire services were obtained. For public transportation, the costs of a return trip for two people was calculated as it was assumed that at least one person would accompany the patient to hospital at every visit because information obtained from the case notes indicated that the patients presented quite late in the course of the disease. For car hire, the cost of hiring the vehicle for a return trip was computed. The average costs (public transportation and car hire) were computed for each patient and the average transportation costs per patient calculated for each disease. (See Appendix 1 for table showing average costs per item for each of the three diseases)

For the purposes of this paper, all costs are reported using 2020 as the base year. The 2017 prices were converted to their 2020 values using the average annual inflation rate reported by the Central Bank of Nigeria (CBN) between 2017 and May, 2020 which was 13.23% (28). The costs were then converted to

For peer review only - http://bmjopen.bmj.com/site/about/guidelines.xhtml

their dollar equivalent using the official CBN June 2020 exchange rate of NGN 360.50: USD1.00 (29) to aid comparison with findings from other studies.

Costs were computed using the formulae below:

(i) Total Direct Cost of Care (DC) for all patients for duration of treatment in days

= Direct medical (DM) + Direct non-medical costs (DN-M)

(ii) Average Direct Cost for all the patients for the duration of treatment in days (ADC)

= DC/number of patients (n)

Ethics Considerations

Ethical approval was obtained from the Oyo State Ethics Committee. Permission to retrieve and review patients' mortality records and case notes was obtained from the University College Hospital management and the heads of the relevant departments. The department heads were assured that information which could identify any patient would not be taken.

Patient and Public Involvement statement

Patients or the public WERE NOT involved in the design, or conduct, or reporting, or dissemination plans of our research

Results

The mean age of the five patients (three male and two female) with a diagnosis of lung cancer was, 59.8±10.6 years. The patients (eight males and one female) with liver cancer had an average age of 52.6 ± 7.9 years; the patients with liver cirrhosis had a mean age of 44.3 ± 13.1 years and all were male.

Medical costs of lung cancer

For peer review only - http://bmjopen.bmj.com/site/about/guidelines.xhtml

Records retrieved revealed that on the average, the patients were managed for 49.2days (from presentation with symptoms to demise) and the average number of days in total spent on admission in hospital was 26.7 days (range: 1 to 90 days). Patients made an average of 1.6 trips to the hospital (range = 1 to 3 days). The average direct medical costs of managing one patient for 49.2 days in 2020 prices was NGN 484,445. 46 (USD 1,343.82) and transportation cost was NGN 25,707.16 (USD 71.31). Average direct (medical and non-medical) cost of managing one patient was about NGN 510, 152.62 (USD 1415.13); (Table 1).

Medical cost of liver cancer

The average number of days in total spent in hospital across all of the patients' admissions was 16.6 days (1 – 77). Details of the average direct costs for managing a patient with liver cancer from (presentation with symptoms to demise) is presented in Table 1. Average direct medical cost was NGN 300.764.15 (USD834.30) per patient and average transportation cost was NGN 8,186.12 (USD22.71), giving average direct medical and non-medical cost of NGN 308,950.27 (USD 857.00).

Medical costs of liver cirrhosis

Details of the average direct costs for managing a patient with liver cirrhosis in 2020 prices is presented in Table 1. The eight patients for whom information was obtained were managed for an average of 21.7 days. The average number of days in total spent in hospital across all of a patient's admissions was 13.9 (1 to 46) days. Average direct medical cost was NGN 235,131.27 (USD 652.24) per patient. Average transportation cost was NGN 2,990.55 (USD 8.30). All the patients were residing in the same town as the hospital at the time of presentation. Total direct medical and non-medical cost of managing one patient with liver cirrhosis was estimated to be NGN 238,121.83 (USD 660.53).

Discussion

This study on the economic costs of NCDs found that all patients for whom records were found were diagnosed in the terminal stages of disease and the average direct medical costs of care were very high. The mean age of the patients who died from lung cancer was 59.8 ± 10.6 years. This is comparable with a mean age of 56.6±21.6 years (range: 30 - 81 years) reported by Ezemba et al. (2012) in a tertiary hospital in South

For peer review only - http://bmjopen.bmj.com/site/about/guidelines.xhtml

East Nigeria (30) and 55 years (range: 40 - 85years) reported by Adewole et al (2017) in a tertiary facility in North Central Nigeria (31). The mean age of patients with liver cancer in our study was 52.6 ± 7.9 years, average number of days spent in hospital across all of a patient's admissions was 16.6 days (range: 1 to 77 days). Patients with liver cirrhosis had a mean age of 44.3 ± 13.1 years, average days on admission and duration of terminal treatment were 13.9 days and 21.7 days respectively. These are similar to a mean age of 46.4 ± 18.0 years reported by Nwokediuko et al (2013) in their review of records of patients with liver disease (including liver cancer and liver cirrhosis) admitted between 2005 and 2010 in a tertiary hospital in South East Nigeria (32). Adekanle et al (2019) similarly documented a mean age of 46.7 ± 15.3 years, mean duration of admission of 33.4 (± 26.5) days and a mortality of 47.6% in their retrospective study of patients with liver disease (including hepatocellular cancer and liver cirrhosis, acute hepatitis and metastatic liver disease among others), conducted between 2013 and 2017in a teaching hospital in Southwest Nigeria (10).

In our study, all the patients for whom data were available presented in the late stages of disease, hence time between presentation and demise was very short. This would have resulted in a reduction in healthcare costs from hospital admission and transportation for follow-up clinic visits and poorer health outcome. Existing literature has highlighted that access and utilization of health care services is still sub-optimal in many developing countries and patients often present late leading to poor disease prognosis (33-35). Many reasons have been given for delayed presentation of patients in hospital. Patient-related reasons for delayed presentation include inadequate knowledge of the disease and of where to obtain appropriate services, preference for traditional providers over orthodox health facilities, poverty and inadequate transportation (34, 36, 37). On the health facility end, experience of the health worker could affect referral for definitive care (36, 37).

The average direct costs (medical and non-medical) for terminal care for each of the diseases studied was very high. We estimated an average direct cost of more than five hundred thousand naira (NGN 510,152.62 i.e. USD 1,415.13) for managing a patient with lung cancer. Similarly, other studies reported high medical

For peer review only - http://bmjopen.bmj.com/site/about/guidelines.xhtml

costs of care for patients with lung cancer. For example, Enstone et al (2018) in their systematic review of the economic burden of small cell lung cancer reported very high direct medical costs (17). Fan et al (2018) reported an average annual direct cost of 63,675 Renminbi (RMB); (USD 10,221, one USD = 6.23 RMB in 2015) per lung cancer patient in China. Patients in their study had been diagnosed up to 2 years prior to the study compared to about 49.2 days in our study. Cipriano et al (2011) reported that monthly costs in the first six months of care for a 72-year old diagnosed with lung cancer in the United States of America in the year 2000, ranged from USD 2,687 (no active treatment) to USD 9,360 (chemo-radiotherapy) in 2006 prices, although these costs varied by stage at diagnosis and histologic type.(19). Sheenan et al (2019) in a more recent study reported an estimate of USD21,603 for a month of care in the terminal phase, for a patient who died of lung cancer at age 70 in 2017 in the United States (18).

In the current study, the average direct cost of managing one patient with liver cancer in Ibadan, Nigeria for approximately 49.2 days in 2020 was estimated to be NGN 308, 950.27 (USD 857.00). Gondek et al (2008) estimated that the annual cost of HCC in the US was approximately $31,641 per patient (16). The average direct cost of managing one patient with liver cirrhosis for less than a month of terminal care (21.7 days) in our study was estimated to be NGN 238,121.83 (USD 660.53) and all patients resided in the hospital town. Although these costs are quite substantial, corresponding costs for patients residing out-of-town would be much higher. This is because out-of-town patients would incur additional non-medical costs from higher inter-city transportation, short-stay accommodation and upkeep for the patient and accompanying person when they come for hospital appointments. Intangible costs from the stress associated with the inter-city travel would also be considerable. In a study of patients with cirrhosis in Vietnem, Nguyen et al (2017) estimated that the median cost of treatment per session in 2015 was 7,439,527.40 ± 5,251,403.97 VND; i.e. approximately USD 329.90±232.88 (1 US Dollar in Vietnamese Dong: USD22,550.18 for 12/31/2015) (38) of which almost 69% of the costs were covered by health insurance (20).

For peer review only - http://bmjopen.bmj.com/site/about/guidelines.xhtml

Although comparisons between our estimates and those provided by the studies cited above need to be made with caution because of our small sample, variations in the disease stage, exact types and duration of treatment provided (among other variables), our findings show that irrespective of the setting, the amount expended to treat each of the diseases considered is significant. Boutayeb and Boutayeb (2005) in their review of the burden of non-communicable diseases in developing countries highlighted the huge difference in costs expended in managing NCDs between patients in developed and developing countries and between affluent and poor patients within developed countries noting that this reflected inequity in access to treatment. In developed countries, a significant proportion of the treatment costs are covered by health insurance and availability of free or largely subsidized care promotes early presentation of patients. This is unlike the situation in Nigeria where the monthly minimum wage for government civil servants was only recently increased to NGN 30,000.00 - less than USD 85.00 in 2019 (39). Treatment costs are also borne out-of-pocket by the generality of the population in Nigeria and many lower income countries where a significant proportion of the population lives on less than a dollar a day and treatment costs are largely borne out-of-pocket. Thus these costs are catastrophic for many families and further explain why many patients present late or default after initial presentation and thus their data does not get captured in formal mortality data.

Conclusion

Our study has demonstrated that direct medical costs of lung and liver cancer and liver cirrhosis in Ibadan, Nigeria are very high, especially when viewed against the prevailing economic environment of the country. Furthermore, many patients in this setting present late resulting in poor prognosis. The substantial costs of care further emphasizes the need for greater efforts at prevention of these diseases. Major risk factors for each of the diseases investigated have been identified and interventions exist to reduce engagement in these risky practices. Furthermore, early diagnosis which can only occur if patients present early and are diagnosed on time needs to be urgently addressed. This would improve disease outcome in the patients and possible reduce medical costs.

For peer review only - http://bmjopen.bmj.com/site/about/guidelines.xhtml

Limitations

1. Use of hospital records would have missed other economic costs including costs incurred in other health facilities while trying to obtain a diagnosis, costs of modifications to housing to make homes conducive for patients, indirect and intangible costs. We acknowledge this constraint and limited costs estimates to direct medical and non-medical (transportation) costs only.
2. The hospital data used to compute the average costs of long-term illness were obtained from a patients' records in their case notes. However, all the case notes could not be retrieved. This was because patient records are yet to be fully computerized, hence the case notes had to be manually retrieved. However, all case notes were not found because some were yet to be returned to the central records office as they were still being processed administratively. In addition, records found were for patients who had an average survival of less than a month indicating late stage disease. This could have resulted in an under-estimation of the overall costs incurred.
3. Transportation costs were based on the initial addresses documented in the patients' case notes and it was not possible to determine if the patients had re-located closer to the hospital during the course of the illness. This could have led to an over-estimation of the costs for the patients who relocated closer to the hospital although costs of relocating to a closer location could offset this difference. The costs however still provide a reasonable estimation of the transportation costs.

4. The hospital is a federal government tertiary facility, however, treatment costs could therefore differ from costs in other federal and private facilities within the country. Medical costs in the private wing of the study hospital are typically at least three times more than the costs in the main stream hospital and consultation fees and admission fees are considerable higher.
5. Some information such as costs for drug supplements, dietary modifications, costs of seeking for additional medical care from other hospitals, costs of repeat out-patient visits, costs of investigations conducted to arrive at a definitive diagnosis and costs of cancer management received (surgical, chemotherapy and/ or radiotherapy) would have been missed.

For peer review only - http://bmjopen.bmj.com/site/about/guidelines.xhtml

Inspite of these limitations, our findings are still very useful and represent estimates of costs of terminal care for lung cancer and liver cancer and cirrhosis in our setting.

Funding statement

This research was supported by the Consortium for Advanced Research Training in Africa (CARTA). CARTA is jointly led by the African Population and Health Research Center and the University of the Witwatersrand and funded by the Carnegie Corporation of New York (Grant No. G-16-54067), Sida (Grant No:54100113), the DELTAS Africa Initiative (Grant No: 107768/Z/15/Z). The DELTAS Africa Initiative is an independent funding scheme of the African Academy of Sciences (AAS)'s Alliance for Accelerating Excellence in Science in Africa (AESA) and supported by the New Partnership for Africa's Development Planning and Coordinating Agency (NEPAD Agency) with funding from the Wellcome Trust (UK) and the UK government. The statements made and views expressed are solely the responsibility of the Fellow.

Acknowledgements

All research staff and respondents, management and heads of relevant departments of the University College Hospital, Ibadan are appreciated.

Competing interests

The authors declare that they have no known competing interests

Authors' contributions

AO conceptualized and developed the protocol for the main study from which the dataset for the current paper was obtained as this formed the research for her doctoral thesis. AO conducted the literature search, and was involved in data collection, analysis and write-up of the current paper. AS and OO were AO's doctoral supervisors. They supervised the entire project and were involved in the write up and revision of the current paper. EA was involved in data collection and write-up of the data for the current paper. TA and

For peer review only - http://bmjopen.bmj.com/site/about/guidelines.xhtml

GO collated the clinical information of all patients who died in the hospital and generated a list of all-cause mortality for patients managed in the teaching hospital. All authors read and approved the final manuscript.

Data sharing statement

No additional data available

References

1. Habib SH, Saha S. Burden of non-communicable disease: Global overview. Diabetes & Metabolic Syndrome: Clinical Research & Reviews. 2010 2010/01/01/;4(1):41-7.
2. World Health Organization. Rethinking “diseases of affluence”the economic impact of chronic diseases. WHO, 2005.
3. Boutayeb A, Boutayeb S. The burden of non communicable diseases in developing countries. International journal for equity in health. 2005 Jan 14;4(1):2. PubMed PMID: 15651987. Pubmed Central PMCID: PMC546417. Epub 2005/01/18. eng.
4. World Health Organization. Health for the world's adolescents: a second chance in the second decade: summary. World Health Organization, 2014.
5. Alwan A, Maclean DR, Riley L, et al. Monitoring and surveillance of chronic non-communicable diseases: progress and capacity in high-burden countries. Lancet. 2010;376:1861 - 68.
6. Beaglehole R, Bonita R, Horton R, Adams C, Alleyne G, Asaria P, et al. Priority actions for the non-communicable disease crisis. Lancet. 2011 Apr 23;377(9775):1438-47. PubMed PMID: 21474174. Epub 2011/04/09. eng.
7. Jaspers L, Colpani V, Chaker L, van der Lee SJ, Muka T, Imo D, et al. The global impact of non-communicable diseases on households and impoverishment: a systematic review. European Journal of Epidemiology. 2015;30(3):163-88.
8. World Health Organization. Noncommunicable Diseases (NCD) Country Profiles2018. Available from: https://www.who.int/nmh/countries/nga_en.pdf?ua=1.
9. The Global Cancer Observatory. Nigeria Factsheet. 2020.
10. Adekanle O, Ijarotimi O, Obasi E, Anthony-Nwojo N, Ndububa D. A Southwest Nigerian tertiary hospital 5-year study of the pattern of liver disease admission. NIGERIAN JOURNAL OF GASTROENTEROLOGY AND HEPATOLOGY. 2020 January 1, 2020;12(1):18-23.
11. Rice D. Cost-of-illness studies: fact or fiction? The Lancet. 1994;344(8936):1519-20.
12. Rice DP. Cost of illness studies: what is good about them? Injury Prevention. 2000;6(3):177-9.
13. Segel JE. Cost-of-illness studies—a primer. RTI-UNC Center of Excellence in Health Promotion Economics. 2006:1-39.
14. Rice DP, Kelman S, Miller LS. Estimates of economic costs of alcohol and drug abuse and mental illness, 1985 and 1988. Public health reports. 1991;106(3):280.
15. World Health Organization. WHO guide to identifying the economic consequences of disease and injury. 2009.
16. Gondek K, Lang K, Danchenko N, Shah S, Anderson S, Thompson D. Economic costs of hepatocellular carcinoma in the United States. Journal of Clinical Oncology. 2008;26(15_suppl):6555-.
17. Enstone A, Greaney M, Povsic M, Wyn R, Penrod JR, Yuan Y. The economic burden of small cell lung cancer: a systematic review of the literature. PharmacoEconomics-open. 2018;2(2):125-39.
18. Sheehan DF, Criss SD, Chen Y, Eckel A, Palazzo L, Tramontano AC, et al. Lung cancer costs by treatment strategy and phase of care among patients enrolled in Medicare. Cancer medicine. 2019

For peer review only - http://bmjopen.bmj.com/site/about/guidelines.xhtml

Jan;8(1):94-103. PubMed PMID: 30575329. Pubmed Central PMCID: PMC6346221. Epub 2018/12/24. eng.
19. Cipriano LE, Romanus D, Earle CC, Neville BA, Halpern EF, Gazelle GS, et al. Lung cancer treatment costs, including patient responsibility, by disease stage and treatment modality, 1992 to 2003. Value Health. 2011;14(1):41-52. PubMed PMID: 21211485. eng.
20. Nguyen D, Nguyen T, Pho N. Analyzing the treatment cost of liver cirrhosis from healthcare payer's perspective of Vietnam in 2015. Value in Health. 2017;20(9):A633.
21. Rezaei S, Akbari Sari A, Arab M, Majdzadeh R, Mohammad Poorasl A. Economic burden of smoking: a systematic review of direct and indirect costs. Med J Islam Repub Iran. 2016;30:397-. PubMed PMID: 27579287. eng.
22. Muka T, Imo D, Jaspers L, Colpani V, Chaker L, van der Lee SJ, et al. The global impact of non-communicable diseases on healthcare spending and national income: a systematic review. European journal of epidemiology. 2015;30(4):251-77.
23. Mutyambizi C, Pavlova M, Chola L, Hongoro C, Groot W. Cost of diabetes mellitus in Africa: a systematic review of existing literature. Globalization and Health. 2018 2018/01/16;14(1):3.
24. Rice D. Estimating the cost of illness: health economics series No. 6, PHS No. 947–6. Washington, DC: US Government Printing Office. 1966.
25. Chisholm D, Stanciole AE, Tan Torres Edejer T, Evans DB. Economic impact of disease and injury: counting what matters. BMJ. 2010;340.
26. Jedy-Agba E, Curado MP, Ogunbiyi O, Oga E, Fabowale T, Igbinoba F, et al. Cancer Incidence in Nigeria: A Report from Population-based Cancer Registries. Cancer epidemiology. 2012 05/22;36(5):e271-e8. PubMed PMID: PMC3438369.
27. Ladep NG, Taylor-Robinson SD. Management of liver disease in Nigeria. Clinical medicine (London, England). 2007 Oct;7(5):439-41. PubMed PMID: 17990704. Pubmed Central PMCID: PMC4953034. Epub 2007/11/10. eng.
28. CBN. Central Bank of Nigeria: Data and Statistics - Inflation rates n.d. [cited 2017 26 July]. Available from: https://www.cbn.gov.ng/rates/inflrates.asp.
29. CBN. Central Bank of Nigeria: Exchange rate archives: Central Bank of Nigeria; n. d. [cited 2017 6 March, 2017]. Available from: http://www.cbn.gov.ng/rates/ExchangeArchives.asp.
30. Ezemba N, Ekpe EE, Eze JC. Challenges of lung cancer management in a developing country. Nigerian journal of medicine: journal of the National Association of Resident Doctors of Nigeria. 2012;21(2):214-7.
31. Adewole OO, Anteyi EA, Dosunmu AE, Ajuwon ZA, Erhabor GE, Betiku YL. P1-009: A clinico pathologic features and Management of Lung cancer in a Nigeria. Journal of Thoracic Oncology. 2007;2(8):S553-S4.
32. Nwokediuko S, Osuala P, Uduma U, Alaneme A, Onwuka C, Mesigo C. Pattern of liver disease admissions in a Nigerian tertiary hospital. Nigerian Journal of Clinical Practice. 2013 July 1, 2013;16(3):339-42.
33. Igwegbe AO, Eleje GU, Ugboaja JO, Ofiaeli RO. Improving maternal mortality at a university teaching hospital in Nnewi, Nigeria. International Journal of Gynecology & Obstetrics. 2012 2012/03/01/;116(3):197-200.
34. Ezeome E. Delays in presentation and treatment of breast cancer in Enugu, Nigeria. Nigerian journal of clinical practice. 2010;13(3).
35. Akande TM, Owayemi J. Health-seeking behaviour in Anyigba, North-Central Nigeria. Res J Med Sci. 2009;3:47-51.
36. Almuammar A, Dryden C, Burr J. Factors associated with late presentation of cancer: a limited literature review. Journal of Radiotherapy in Practice. 2010;9(2):117.
37. Idowu OE, Apemiye RA. Delay in presentation and diagnosis of adult primary intracranial neoplasms in a tropical teaching hospital: A pilot study. International Journal of Surgery. 2009 2009/01/01/;7(4):396-8.

For peer review only - http://bmjopen.bmj.com/site/about/guidelines.xhtml

38. rates.org. E. [20 June, 2020]. Available from: https://www.exchange-rates.org/Rate/USD/VND/12-31-2015.
39. Nwanne C. New minimum wage of N30, 000 takes effect. The Guardian. 2019.

For peer review only

Table 1: Average direct medical and transportation costs for managing patients who died from selected NCDs in 2020

Duration of treatment (Days)	Average No. of hospital visit (appointments)	Average No. of days on admission	Average direct medical costs per person	Average direct non-medical costs (transportation)	Average direct costs per person
**A**	**B**	**C**	**D**	**E**	**F = D + E**
Lung Ca					

For peer review only - http://bmjopen.bmj.com/site/about/guidelines.xhtml

49.2	1.6	26.7	NGN 484, 445.46<br>USD 1,343.82	NGN 25,707.16<br>USD 71.31	NGN 510,152.62<br>USD 1,415.13
Liver Ca*					
16.6	1	16.6	NGN 300,764.15<br>USD 834.30	NGN 8,186.12<br>USD 22.71	NGN 308,950.27<br>USD 857.00
Liver cirrhosis					
21.7	1	13.9	NGN 235,131.27<br>USD 652.24	NGN 2,990.55<br>USD 8.30	NGN 238,121.83<br>USD 660.53

*Duration on treatment (management) for lung ca was the same as days on admission for all patients whose records were found because they were all admitted on the day of presentation and stayed on admission until demise.

For peer review only

Appendix 1: Average costs (2020 prices) per patient for a month of terminal care for patients with lung cancer, liver cancer and liver cirrhosis

	Admission history (average)			Average costs for patients										
	Average times of admission	Average hospital visits made	Average days on Admission *	Registration	Consultation	Service Charge	Drugs*	Consumables	Admission	Investigations	Bed side Procedures**	Average Direct Medical	Average Direct Non-medical (transport)	Average Direct medical and non-medical
**Lung Ca (n = 5)**														
2020 naira	1.2	1.6	27.4	1959.83	4442.28	1306.55	13128.24	17798.16	39486.94	354061.35	52262.12	484445.46	25707.16[a]	510152.62
2020 dollar				5.44	12.32	3.62	36.42	49.37	109.53	982.14	144.97	1343.82	71.31	1415.13
**Liver Ca (n = 9)**														
2020 naira	1	1	17.1	1959.83	2467.93	725.86	14404.02	36853.50	24840.48	193381.46	26131.06	300764.15	8186.12[b]	308950.27
2020 dollar				5.44	6.85	2.01	39.96	102.23	68.91	536.43	72.49	834.30	22.71	857.00
**Liver cirrhosis (n = 8)**														
2020 naira	1	1.25	14.6	1959.83	3084.92	907.33	24231.84	18282.67	21231.49	139302.14	26131.06	235131.27	2990.55[c]	238121.82
2020 dollar				5.44	8.56	2.52	67.22	50.71	58.89	386.41	72.49	652.24	8.30	660.53

* Average number of days in total spent in hospital across all of a patient's admissions

a= 4 patients came from other states b = 3 patients came from another state, c =1 patient came from another state

*Drugs listed were mainly supportive for most of the patients

**Examples of bedside procedures - CTTD: Closed tube thoracostomy drainage, liver biopsy etc.

For peer review only

STROBE Statement—checklist of items that should be included in reports of observational studies

	Item No	Recommendation
**Title and abstract**	1	(*a*) Indicate the study's design with a commonly used term in the title or the abstract
		(*b*) Provide in the abstract an informative and balanced summary of what was done and what was found
		Author comment: Abstract included in line with journal specifications Page 3 of 24
**Introduction**		
Background/rationale	2	Explain the scientific background and rationale for the investigation being reported
		Author comment: Information in Background of paper – Pages 5 and 6 of 24
Objectives	3	State specific objectives, including any prespecified hypotheses
		Author comment: Information is in the concluding paragraph of the introduction, just before the results.- Page 6 of 24
**Methods**		
Study design	4	Present key elements of study design early in the paper
		Author comment: Information in opening paragraph of the methods –Page 7 of 24
Setting	5	Describe the setting, locations, and relevant dates, including periods of recruitment, exposure, follow-up, and data collection
		Author comment: In methods – paragraphs 2 and 3 – Page 7 of 24
Participants	6	(*a*) *Cohort study*—Give the eligibility criteria, and the sources and methods of selection of participants. Describe methods of follow-up<br>*Case-control study*—Give the eligibility criteria, and the sources and methods of case ascertainment and control selection. Give the rationale for the choice of cases and controls<br>*Cross-sectional study*—Give the eligibility criteria, and the sources and methods of selection of participants<br>Author comment: Information in paragraph 3 of methods Page 7 of 24
		(*b*) *Cohort study*—For matched studies, give matching criteria and number of exposed and unexposed<br>*Case-control study*—For matched studies, give matching criteria and the number of controls per case
Variables	7	Clearly define all outcomes, exposures, predictors, potential confounders, and effect modifiers. Give diagnostic criteria, if applicable<br>Author comment: Paragraphs 3, 4 and 5 of the methods section on pages 7 to 8 of 24
Data sources/ measurement	8*	For each variable of interest, give sources of data and details of methods of assessment (measurement). Describe comparability of assessment methods if there is more than one group<br>Author comment Paragraphs 3, 4 and 5 of the methods section on pages 7 to 8 of 24
Bias	9	Describe any efforts to address potential sources of bias<br>Author comment: Medical and transportation costs were obtained from at least two sources. Information presented in Pages 7 and 8 of 24
Study size	10	Explain how the study size was arrived at<br>Author comment: Primary data was not collected and all available records found were analysed.
Quantitative variables	11	Explain how quantitative variables were handled in the analyses. If applicable, describe which groupings were chosen and why

For peer review only

		Author comment: Information on variables is provided in the section just preceding the ethical approval section Pages 7 to 9 of 24
Statistical methods	12	(*a*) Describe all statistical methods, including those used to control for confounding
		(*b*) Describe any methods used to examine subgroups and interactions
		(*c*) Explain how missing data were addressed
		(*d*) *Cohort study*—If applicable, explain how loss to follow-up was addressed<br>*Case-control study*—If applicable, explain how matching of cases and controls was addressed<br>*Cross-sectional study*—If applicable, describe analytical methods taking account of sampling strategy
		(*e*) Describe any sensitivity analyses
		Author comment: Information presented in the methods section – the sub-section just preceding the results Pages 8 and 9 of 24

Continued on next page

For peer review only

**Results**		
Participants	13*	(a) Report numbers of individuals at each stage of study—eg numbers potentially eligible, examined for eligibility, confirmed eligible, included in the study, completing follow-up, and analysed - Methods section Page 7 of 24 second paragraph
		(b) Give reasons for non-participation at each stage
		(c) Consider use of a flow diagram
Descriptive data	14*	(a) Give characteristics of study participants (eg demographic, clinical, social) and information on exposures and potential confounders
		(b) Indicate number of participants with missing data for each variable of interest
		(c) *Cohort study*—Summarise follow-up time (eg, average and total amount)<br>Page 10. Opening paragraph of the results section: Page 10 of 24
Outcome data	15*	*Cohort study*—Report numbers of outcome events or summary measures over time
		Case-control study—Report numbers in each exposure category, or summary measures of exposure
		Cross-sectional study—Report numbers of outcome events or summary measures
Main results	16	(*a*) Give unadjusted estimates and, if applicable, confounder-adjusted estimates and their precision (eg, 95% confidence interval). Make clear which confounders were adjusted for and why they were included
		(*b*) Report category boundaries when continuous variables were categorized
		(*c*) If relevant, consider translating estimates of relative risk into absolute risk for a meaningful time period<br>Not applicable
Other analyses	17	Report other analyses done—eg analyses of subgroups and interactions, and sensitivity analyses
		Author comment: Information presented in the results section – Pages 10 to 11 of 24
**Discussion**		**Author comment: Information presented in the results section – Pages 11 to 13 of 24**
Key results	18	Summarise key results with reference to study objectives
Limitations	19	Discuss limitations of the study, taking into account sources of potential bias or imprecision. Discuss both direction and magnitude of any potential bias<br>Author comment: Information presented after the concluding sub-section –Pages 13 and 14 of 24
Interpretation	20	Give a cautious overall interpretation of results considering objectives, limitations, multiplicity of analyses, results from similar studies, and other relevant evidence<br>Author comment: Information provided in the discussion section - Pages 11 to 13 of 24
Generalisability	21	Discuss the generalisability (external validity) of the study results
		Author comment: Information presented in the conclusion section Page 13 of 24
**Other information**		
Funding	22	Give the source of funding and the role of the funders for the present study and, if applicable, for the original study on which the present article is based<br>Author comment: Information presented after the limitations Page 14 of 24

*Give information separately for cases and controls in case-control studies and, if applicable, for exposed and unexposed groups in cohort and cross-sectional studies.

Note: An Explanation and Elaboration article discusses each checklist item and gives methodological background and published examples of transparent reporting. The STROBE checklist is best used in conjunction with this article (freely available on the Web sites of PLoS Medicine at http://www.plosmedicine.org/, Annals of Internal Medicine at

For peer review only - http://bmjopen.bmj.com/site/about/guidelines.xhtml

http://www.annals.org/, and Epidemiology at http://www.epidem.com/). Information on the STROBE Initiative is available at www.strobe-statement.org.

For peer review only

BMJ Open

Economic costs of terminal care for selected non-communicable diseases from a healthcare perspective: A retrospective review of mortality records from a tertiary hospital in Nigeria

Journal:	*BMJ Open*
Manuscript ID	bmjopen-2020-044969.R2
Article Type:	Original research
Date Submitted by the Author:	23-Mar-2021
Complete List of Authors:	Olumide, Adesola; University of Ibadan College of Medicine, Institute of Child Health<br>Shmueli, Amir; Hebrew University of Jerusalem Braun School of Public Health and Community Medicine, School of Public Health,<br>Omotade, Olayemi; University of Ibadan, College of Medicine, Institute of Child Health<br>Adebayo, Emmanuel; University of Ibadan College of Medicine, Institute of Child Health<br>Alonge, Temitope; University of Ibadan College of Medicine, Surgery<br>Ogun, Gabriel; University of Ibadan College of Medicine, Pathology
**Primary Subject Heading**:	Public health
Secondary Subject Heading:	Health economics
Keywords:	PUBLIC HEALTH, PREVENTIVE MEDICINE, Health policy < HEALTH SERVICES ADMINISTRATION & MANAGEMENT, Health economics < HEALTH SERVICES ADMINISTRATION & MANAGEMENT

SCHOLARONE™
Manuscripts

I, the Submitting Author has the right to grant and does grant on behalf of all authors of the Work (as defined in the below author licence), an exclusive licence and/or a non-exclusive licence for contributions from authors who are: i) UK Crown employees; ii) where BMJ has agreed a CC-BY licence shall apply, and/or iii) in accordance with the terms applicable for US Federal Government officers or employees acting as part of their official duties; on a worldwide, perpetual, irrevocable, royalty-free basis to BMJ Publishing Group Ltd ("BMJ") its licensees and where the relevant Journal is co-owned by BMJ to the co-owners of the Journal, to publish the Work in this journal and any other BMJ products and to exploit all rights, as set out in our licence.

The Submitting Author accepts and understands that any supply made under these terms is made by BMJ to the Submitting Author unless you are acting as an employee on behalf of your employer or a postgraduate student of an affiliated institution which is paying any applicable article publishing charge ("APC") for Open Access articles. Where the Submitting Author wishes to make the Work available on an Open Access basis (and intends to pay the relevant APC), the terms of reuse of such Open Access shall be governed by a Creative Commons licence – details of these licences and which Creative Commons licence will apply to this Work are set out in our licence referred to above.

Other than as permitted in any relevant BMJ Author's Self Archiving Policies, I confirm this Work has not been accepted for publication elsewhere, is not being considered for publication elsewhere and does not duplicate material already published. I confirm all authors consent to publication of this Work and authorise the granting of this licence.

For peer review only

Economic costs of terminal care for selected non-communicable diseases from a healthcare perspective: A retrospective review of mortality records from a tertiary hospital in Nigeria

* Adesola O Olumide.[1], Amir Shmueli [2], Olayemi O. Omotade[1], Emmanuel S Adebayo [3], Temitope O. Alonge[4], Gabriel O. Ogun [5]

1. Institute of Child Health, College of Medicine, University of Ibadan and University College Hospital, Ibadan, Nigeria
2. School of Public Health, Hebrew University of Jerusalem, Israel
3. Institute of Child Health, College of Medicine, University of Ibadan Nigeria
4. Department of Surgery, College of Medicine, University of Ibadan and University College Hospital, Ibadan, Nigeria
5. Department of Pathology, College of Medicine, University of Ibadan and University College Hospital, Ibadan, Nigeria

*Corresponding author: Adesola O. Olumide, Institute of Child Health, College of Medicine, University of Ibadan and University College Hospital, Ibadan, Nigeria

Email: daisyolu@yahoo.co.uk; ; aoolumide@com.ui.edu.ng

Short title: Economic costs of terminal care for selected NCDs in Nigeria

Key words: Economic costs; Non-communicable disease; Lung CA; Liver Ca; Liver cirrhosis; Medical costs;

For peer review only - http://bmjopen.bmj.com/site/about/guidelines.xhtml

Economic costs of terminal care for selected non-communicable diseases from a healthcare perspective: A retrospective review of mortality records from a tertiary hospital in Nigeria

Abstract

Introduction: The World Health Organization revealed that morbidity and mortality from Non-Communicable Diseases (NCDs) are on the increase and NCDs accounted for approximately 29% of all deaths in Nigeria in 2016. This study was conducted to estimate the economic cost of selected non-communicable diseases – lung cancer, liver cancer and liver cirrhosis. These diseases are known to be associated with key modifiable health-risk behaviours (smoking and alcohol use) which are prevalent in Nigeria and often commence during the adolescent years.

Methods: Data were obtained between 2016 and 2017, from mortality records of patients managed for the selected diseases in the University College Hospital, a major referral center in Nigeria. Information on costs of treatment, clinic visits, admission etc. and transportation were obtained. Average costs of terminal in-hospital care and transportation costs (in 2020 prices) were computed per patient. Costs were converted to the United States (US) dollar equivalent using the current official rate of USD 1: Nigerian Naira (NGN) 360.50.

Results: Twenty-two (out of 90 cases recorded) could be retrieved and all the patients had been diagnosed in the terminal stages of the disease. The average direct costs were NGN510, 152.62 (USD 1415.13) for an average of 49.2 days of terminal care for lung cancer; NGN 308,950.27 (USD 857.00) and NGN 238,121.83 (USD 660.53) for an average of 16.6 and 21.7 days of terminal care for patients managed for liver cancer and liver cirrhosis respectively.

Conclusion: The economic costs of each of the diseases were very high. Findings emphasize the need for aggressive efforts to promote primary prevention, improve early diagnosis and provide affordable treatment in view of the fact that the monthly minimum wage is less than USD 85.00 and treatment costs are borne out-of-pocket by the generality of the population in Nigeria.

For peer review only - http://bmjopen.bmj.com/site/about/guidelines.xhtml

Article summary

Strengths and limitations of this study

- This is a novel study providing direct costs of terminal care for selected Non-Communicable Diseases (NCDs) in Nigeria from a healthcare perspective
- The data collection methods utilized were robust; lending credence to the strength of the findings
- Costs are based on data retrieved from a Federal government tertiary hospital in Nigeria which are lower than equivalent costs obtainable in private facilities.
- Cost could only be computed for the 22 records retrieved (representing 24.4% of all related mortalities within the time-frame studied)
- In spite of the small sample, the findings are reasonable estimates of direct costs of terminal care for the selected NCDs in the study setting and are an important first step to further enquiry.

Keywords

Economic costs; Noncommunicable disease; Medical costs; Lung cancer, Liver cancer, Liver cirrhosis

Introduction

Globally, there has been an increase in the prevalence of non-communicable diseases (1). These diseases were previously viewed as diseases of the affluent with a high prevalence in developed countries (2). This is no longer the case however and the larger burden of these diseases is now borne by low and middle income countries(1). While still grappling with the burden from communicable diseases like malaria and pneumonia, developing countries are experiencing an increase in the occurrence of chronic non-communicable diseases (1, 3). Thus, these countries are currently experiencing a double burden of diseases from communicable and non-communicable diseases (1, 3). Research has proven that many of the chronic conditions that individuals suffer in adulthood are linked to unhealthy habits e.g. substance including tobacco and alcohol use and unhealthy eating habits and unsafe sexual intercourse that they adopted during their younger years (4-6). Common established outcomes of these health-risk behaviours include the occurrence of various cancers and other non-communicable conditions such as cardiovascular diseases and

For peer review only - http://bmjopen.bmj.com/site/about/guidelines.xhtml

diabetes later in life (1). These diseases result in considerable morbidity, mortality and economic losses (7) emphasizing the need for continued research to inform policy and practice geared towards their control.

In Nigeria the disease burden from communicable diseases is still very high, however, available data indicate that the burden from Non-Communicable diseases is significant. The World Health Organization estimated that morbidity and mortality from NCDs accounted for approximately 29% of all deaths in the country in 2016 (8). According to 2020 data from the Global cancer observatory, there were about 124,815 new cases of cancer in Nigeria. Liver and lung cancer were among the top 20 causes of cancer in Nigeria with incidence rates of 4.4% and 1.4% respectively (based on weighted/simple average of the most recent local rates applied to 2020 population) (9). Furthermore of the estimated 78,899 deaths from cancer, 5,180 and 1,789 were from liver and lung cancer respectively. Adekanle, et al (2020) in their review of records of patients admitted with liver diseases into a tertiary hospital in Ile‑Ife, Nigeria, from 2013–2017 found that hepatocellular carcinoma and liver cirrhosis accounted for 52.8% and 27.2% of all cases of liver disease admitted during the review period (10).

Cost of illness studies determine the burden of diseases to society in monetary terms. In addition, they provide an estimate of the amount that could potentially be saved if a disease were to be eradicated (11-13). Typically, cost of illness studies do not evaluate interventions that address the disease under study. Thus they do not provide information on the benefits of implementing specific interventions or which of a range of interventions would produce maximal impact (13). Cost of illness studies as the name implies, document a range of illness costs which are usually categorized as direct, indirect or intangible costs. Direct costs measure, “the opportunity cost of resources used for treating a particular illness” or, “costs for which payments are actually made”. Examples of direct costs could be direct medical costs such as costs of treatment, drugs, in-patient admission, etc. or direct non-medical costs such as transportation costs, costs of special meals, relocation costs etc. (13, 14). Indirect costs refer to, “the value of resources lost due to a particular illness”, and intangible costs include costs of pain and suffering (13, 14).

For peer review only - http://bmjopen.bmj.com/site/about/guidelines.xhtml

There has been an increase in the numbers of studies documenting the cost of various non-communicable diseases, however, the overwhelming majority of these studies have been conducted in developed countries or in developing countries in Asia (15-21). Data from existing studies confirm the immense economic burden from Non-Communicable diseases. Muka et al (2015) in their review of the global impact of non-communicable diseases on healthcare found that globally, cancer and cardiovascular diseases (CVD) had the highest reported mean annual total direct costs per patient of approximately 197,772 USD and 81,096 USD, respectively. (22) A systematic review of cost of diabetes mellitus in Africa by Mutyambizi et al (2018) reported national direct costs of diabetes ranging from 3.5 to 4.5 billion International dollars per annum (23). There remains a paucity of these studies in Sub-Saharan African and in Nigeria, resulting in a reliance on cost estimates from developed countries or developing countries outside of sub-Saharan Africa for drawing inferences for the economic burden of diseases in our region. The current study was conducted to determine the cost of selected chronic illnesses, namely, lung cancer, liver cancer and chronic liver disease in Ibadan Nigeria. These illnesses were selected because they are recognized long term consequences of some modifiable health-risk behaviours associated with a significant proportion of NCDs, namely smoking and alcohol consumption, they result in significant morbidity and mortality and there are proven measures for primary prevention of the health-risk behaviours they are associated with. Data were obtained as part of a larger study on the predictors and economic costs of selected health-risk behaviours among adolescents in Oyo state, Nigeria.

Methods

This prevalence-based study adopted a health care system perspective and thus focused on the medical costs of the illnesses. The estimation of the cost of care for the long-term illnesses in this study was guided by the cost-of-illness approach described by Rice (24) and the WHO economic framework of cost estimation

For peer review only

described in the World Health Organization Guide to identifying the economic consequences of disease and injury (15, 25)

Data on the illness costs were collected between September 2016 and January, 2017 from the University College Hospital (UCH), Ibadan. Established in 1952, it is the premier teaching hospital in Nigeria and is located in Ibadan, South west Nigeria. The hospital also houses the first cancer registry in Nigeria - the Ibadan Cancer Registry which was established in 1960 within the Pathology department of the University of Ibadan (26). The UCH is a training centre for doctors, nurses and various other categories of health staff at undergraduate and postgraduate levels and also provides tertiary level health services. It is a major receiving centre for tertiary level management of patients with various communicable and non-communicable diseases who are referred from various parts of the country.

We obtained data retrospectively from a review of case records of patients who had been diagnosed and received treatment for the illnesses investigated. The Case note numbers of patients who died from the diseases of interest – lung cancer and liver disease (liver cancer and liver cirrhosis) in 2014 and 2015 were obtained from the Medical records department. There was a total of 12 reported deaths from lung cancer, of these, 5 case notes could be retrieved. In all, 78 patients were reported to have died from liver diseases (liver cirrhosis, liver cancer and chronic liver disease) in 2014 and 2015. Of these, nine case notes of patients with liver cancer and eight for patients with liver cirrhosis could be retrieved. Nine casefiles of patients with chronic liver disease were retrieved but information from these cases notes were not utilized because there was no definitive diagnosis in the case note (this could have occurred because payment was yet to be made for the investigations, hence they were carried out). Ladep et al (2007) had noted that some other challenges to a definitive diagnosis of liver cirrhosis in Nigeria which were reluctance to give consent for liver biopsy and inadequate trained doctors to conduct the procedure (27). Relevant information – age, sex, home address, date of diagnosis, medical treatment, investigations, number of clinic visits, admission history (number of admissions and duration on admission) etc. were extracted manually from the patient case notes onto a datasheet.

For peer review only - http://bmjopen.bmj.com/site/about/guidelines.xhtml

Direct medical (healthcare) costs obtained included the following: cost of hospital admission, clinic visits, investigations, medical procedures, medications, and consumables. The time from presentation with symptoms to demise for the patients whose case records were obtained ranged from zero to about four and a half months indicating that most patients were admitted at the point when the illnesses were at the terminal stages. Costs were obtained from the relevant departments in the hospital. In addition, costs of investigations and drugs were also obtained from a registered private laboratory and a pharmacy within Ibadan. This was because anecdotal reports indicated that some patients procure their drugs or get some of their investigations done outside the hospital. The costs of drug and investigations from these private sources were compared with hospital costs, summed and the average for each item was documented. For each illness investigated (lung cancer, liver cancer, and liver cirrhosis), the total medical costs for each patient was summed and the average costs incurred from the time of diagnosis to demise was estimated for each disease based on information obtained from the patients' case note.

The direct non-medical cost of illness was limited to transportation costs only. Transportation costs were computed for each patient using the home address provided in the case note. A list of all the addresses was compiled and information on transportation costs from these neighbourhoods to the hospital was obtained from informal interviews with commercial transport drivers in the relevant motor-parks. The cost of regular public transportation and the cost of car hire services were obtained. For public transportation, the costs of a return trip for two people was calculated as it was assumed that at least one person would accompany the patient to hospital at every visit because information obtained from the case notes indicated that the patients presented quite late in the course of the disease. For car hire, the cost of hiring the vehicle for a return trip was computed. The average costs (public transportation and car hire) were computed for each patient and the average transportation costs per patient calculated for each disease. (See Appendix 1 for table showing average costs per item for each of the three diseases)

For peer review only - http://bmjopen.bmj.com/site/about/guidelines.xhtml

For the purposes of this paper, all costs are reported using 2020 as the base year. The 2017 prices were converted to their 2020 values using the average annual inflation rate reported by the Central Bank of Nigeria (CBN) between 2017 and May, 2020 which was 13.23% (28). The costs were then converted to their dollar equivalent using the official CBN June 2020 exchange rate of NGN 360.50: USD1.00 (29) to aid comparison with findings from other studies.

Costs were computed using the formulae below:

(i) Total Direct Cost of Care (DC) for all patients for duration of treatment in days

= Direct medical (DM) + Direct non-medical costs (DN-M)

(ii) Average Direct Cost for all the patients for the duration of treatment in days (ADC)

= DC/number of patients (n)

Ethics Considerations

Ethical approval was obtained from the Oyo State Ethics Committee (AD13/479/193). Permission to retrieve and review patients' mortality records and case notes was obtained from the University College Hospital management and the heads of the relevant departments. The department heads were assured that information which could identify any patient would not be taken.

Patient and Public Involvement statement

Patients or the public WERE NOT involved in the design, or conduct, or reporting, or dissemination plans of our research

Results

The mean age (and standard deviation) of the five patients (three male and two female) with a diagnosis of lung cancer was, 59.8 (±10.6) years. The patients (eight males and one female) with liver cancer had an

For peer review only - http://bmjopen.bmj.com/site/about/guidelines.xhtml

average age of 52.6 years (standard deviation = 7.9). There were eight male patients with liver cirrhosis and they had a mean age of 44.3 years (standard deviation = 13.1).

Medical costs of lung cancer

Records retrieved revealed that on the average, the patients were managed for 49.2days (from presentation with symptoms to demise) and the average number of days in total spent on admission in hospital was 26.7 days (range: 1 to 90 days). Patients made an average of 1.6 trips to the hospital (range = 1 to 3 days). The average direct medical costs of managing one patient for 49.2 days in 2020 prices was NGN 484,445. 46 (USD 1,343.82) and transportation cost was NGN 25,707.16 (USD 71.31). Average direct (medical and non-medical) cost of managing one patient was about NGN 510, 152.62 (USD 1415.13); (Table 1).

Medical cost of liver cancer

The average number of days in total spent in hospital across all of the patients' admissions was 16.6 days (1 – 77). Details of the average direct costs for managing a patient with liver cancer from (presentation with symptoms to demise) is presented in Table 1. Average direct medical cost was NGN 300.764.15 (USD834.30) per patient and average transportation cost was NGN 8,186.12 (USD22.71), giving average direct medical and non-medical cost of NGN 308,950.27 (USD 857.00).

Medical costs of liver cirrhosis

Details of the average direct costs for managing a patient with liver cirrhosis in 2020 prices is presented in Table 1. The eight patients for whom information was obtained were managed for an average of 21.7 days. The average number of days in total spent in hospital across all of a patient's admissions was 13.9 (1 to 46) days. Average direct medical cost was NGN 235,131.27 (USD 652.24) per patient. Average transportation cost was NGN 2,990.55 (USD 8.30). All the patients were residing in the same town as the hospital at the time of presentation. Total direct medical and non-medical cost of managing one patient with liver cirrhosis was estimated to be NGN 238,121.83 (USD 660.53).

Discussion

For peer review only - http://bmjopen.bmj.com/site/about/guidelines.xhtml

This study on the economic costs of NCDs found that all patients for whom records were found were diagnosed in the terminal stages of disease and the average direct medical costs of care were very high. The mean age of the patients who died from lung cancer was 59.8 (standard deviation = 10.6) years. This is comparable with a mean age of 56.6 (standard deviation = 21.6) years and range of 30 - 81 years) reported by Ezemba et al. (2012) in a tertiary hospital in South East Nigeria (30) and 55 years (range: 40 - 85years) reported by Adewole et al (2017) in a tertiary facility in North Central Nigeria (31). The mean age of patients with liver cancer in our study was 52.6 (standard deviation = 7.9) years, average number of days spent in hospital across all of a patient's admissions was 16.6 days (range: 1 to 77 days). Patients with liver cirrhosis had a mean age of 44.3 (standard deviation = 13.1) years, average days on admission and duration of terminal treatment were 13.9 days and 21.7 days respectively. These are similar to a mean age of 46.4 (standard deviation = 18.0) years reported by Nwokediuko et al (2013) in their review of records of patients with liver disease (including liver cancer and liver cirrhosis) admitted between 2005 and 2010 in a tertiary hospital in South East Nigeria (32). Adekanle et al (2019) similarly documented a mean age of 46.7 (standard deviation = 15.3) years, mean duration of admission of 33.4 (standard deviation = 26.5) days and a mortality of 47.6% in their retrospective study of patients with liver disease (including hepatocellular cancer and liver cirrhosis, acute hepatitis and metastatic liver disease among others), conducted between 2013 and 2017in a teaching hospital in Southwest Nigeria (10).

In our study, all the patients for whom data were available presented in the late stages of disease, hence time between presentation and demise was very short. This would have resulted in a reduction in healthcare costs from hospital admission and transportation for follow-up clinic visits and poorer health outcome. Existing literature has highlighted that access and utilization of health care services is still sub-optimal in many developing countries and patients often present late leading to poor disease prognosis (33-35). Many reasons have been given for delayed presentation of patients in hospital. Patient-related reasons for delayed presentation include inadequate knowledge of the disease and of where to obtain appropriate services, preference for traditional providers over orthodox health facilities, poverty and inadequate transportation

For peer review only - http://bmjopen.bmj.com/site/about/guidelines.xhtml

(34, 36, 37). On the health facility end, experience of the health worker could affect referral for definitive care (36, 37).

The average direct costs (medical and non-medical) for terminal care for each of the diseases studied was very high. We estimated an average direct cost of more than five hundred thousand naira (NGN 510,152.62 i.e. USD 1,415.13) for managing a patient with lung cancer. Similarly, other studies have reported high medical costs of care for patients with lung cancer. For example, Edis and Karlikaya (2007) reported that among patients in Turkey, the mean total direct costs per lung cancer patient was more than USD 14,000 with costs per patient ranging from USD 771.00 to USD 104,079 (38). Fan et al (2018) reported an average annual direct cost of 63,675 Renminbi (RMB); (USD 10,221, one USD = 6.23 RMB in 2015) per lung cancer patient in China. Patients in their study had been diagnosed up to 2 years prior to the study compared to about 49.2 days in our study. Cipriano et al (2011) reported that monthly costs in the first six months of care for a 72-year old diagnosed with lung cancer in the United States of America in the year 2000, ranged from USD 2,687 (no active treatment) to USD 9,360 (chemo-radiotherapy) in 2006 prices, although these costs varied by stage at diagnosis and histologic type (19). Sheenan et al (2019) in a more recent study reported an estimate of USD21,603 for a month of care in the terminal phase, for a patient who died of lung cancer at age 70 in 2017 in the United States (18).

In the current study, the average direct cost of managing one patient with liver cancer in Ibadan, Nigeria for approximately 49.2 days in 2020 was estimated to be NGN 308, 950.27 (USD 857.00). Gondek et al (2008) estimated that the annual cost of HCC in the US was approximately $31,641 per patient (16). The average direct cost of managing one patient with liver cirrhosis for less than a month of terminal care (21.7 days) in our study was estimated to be NGN 238,121.83 (USD 660.53) and all patients resided in the hospital town. Although these costs are quite substantial, corresponding costs for patients residing out-of-town would be much higher. This is because out-of-town patients would incur additional non-medical costs from higher inter-city transportation, short-stay accommodation and upkeep for the patient and accompanying person when they come for hospital appointments. Intangible costs from the stress associated

For peer review only - http://bmjopen.bmj.com/site/about/guidelines.xhtml

with the inter-city travel would also be considerable. In a study of patients with cirrhosis in Vietnem, Nguyen et al (2017) estimated that the median cost of treatment per session in 2015 was 7,439,527.40 ± 5,251,403.97 VND; i.e. approximately USD 329.90±232.88 (1 US Dollar in Vietnamese Dong: USD22,550.18 for 12/31/2015) (39) of which almost 69% of the costs were covered by health insurance (20).

Although comparisons between our estimates and those provided by the studies cited above need to be made with caution because of our small sample, variations in the disease stage, exact types and duration of treatment provided (among other variables), our findings show that irrespective of the setting, the amount expended to treat each of the diseases considered is significant. Boutayeb and Boutayeb (2005) in their review of the burden of non-communicable diseases in developing countries highlighted the huge difference in costs expended in managing NCDs between patients in developed and developing countries and between affluent and poor patients within developed countries noting that this reflected inequity in access to treatment. In developed countries, a significant proportion of the treatment costs are covered by health insurance and availability of free or largely subsidized care promotes early presentation of patients. This is unlike the situation in Nigeria where the monthly minimum wage for government civil servants was only recently increased to NGN 30,000.00 - less than USD 85.00 in 2019 (40). Treatment costs are also borne out-of-pocket by the generality of the population in Nigeria and many lower income countries where a significant proportion of the population lives on less than a dollar a day and treatment costs are largely borne out-of-pocket. Thus these costs are catastrophic for many families and further explain why many patients present late or default after initial presentation and thus their data does not get captured in formal mortality data.

Conclusion

Our study has demonstrated that direct medical costs of lung and liver cancer and liver cirrhosis in Ibadan, Nigeria are very high, especially when viewed against the prevailing economic environment of the country. Furthermore, many patients in this setting present late resulting in poor prognosis. The substantial costs of

For peer review only - http://bmjopen.bmj.com/site/about/guidelines.xhtml

care further emphasizes the need for greater efforts at prevention of these diseases. Major risk factors for each of the diseases investigated have been identified and interventions exist to reduce engagement in these risky practices. Furthermore, early diagnosis which can only occur if patients present early and are diagnosed on time needs to be urgently addressed. This would improve disease outcome in the patients and possible reduce medical costs.

Limitations

1. The results are based on a relatively small sample, however, they are an important first step to further enquiry.
2. Use of hospital records would have missed other economic costs including costs incurred in other health facilities while trying to obtain a diagnosis, costs of modifications to housing to make homes conducive for patients, indirect and intangible costs. We acknowledge this constraint and limited costs estimates to direct medical and non-medical (transportation) costs only.
3. The hospital data used to compute the average costs of long-term illness were obtained from a patients' records in their case notes. However, all the case notes could not be retrieved. This was because patient records are yet to be fully computerized, hence the case notes had to be manually retrieved. However, all case notes were not found because some were yet to be returned to the central records office as they were still being processed administratively. In addition, records found were for patients who had an average survival of less than a month indicating late stage disease. This could have resulted in an under-estimation of the overall costs incurred.
4. Transportation costs were based on the initial addresses documented in the patients' case notes and it was not possible to determine if the patients had re-located closer to the hospital during the course of the illness. This could have led to an over-estimation of the costs for the patients who relocated closer to the hospital although costs of relocating to a closer location could offset this difference. The costs however still provide a reasonable estimation of the transportation costs.

For peer review only - http://bmjopen.bmj.com/site/about/guidelines.xhtml

5. The hospital is a federal government tertiary facility, however, treatment costs could therefore differ from costs in other federal and private facilities within the country. **Information obtained from expert opinion revealed that**, medical costs in the private wing of the study hospital are typically at least three times more than the costs in the main stream hospital and consultation fees and admission fees are considerable higher.
6. Some information such as costs for drug supplements, dietary modifications, costs of seeking for additional medical care from other hospitals, costs of repeat out-patient visits, costs of investigations conducted to arrive at a definitive diagnosis and costs of cancer management received (surgical, chemotherapy and/ or radiotherapy) would have been missed.

Inspite of these limitations, our findings are still very useful and represent estimates of costs of terminal care for lung cancer and liver cancer and cirrhosis in our setting.

Funding statement

This research was supported by the Consortium for Advanced Research Training in Africa (CARTA). CARTA is jointly led by the African Population and Health Research Center and the University of the Witwatersrand and funded by the Carnegie Corporation of New York (Grant No. G-16-54067), Sida (Grant No:54100113), the DELTAS Africa Initiative (Grant No: 107768/Z/15/Z). The DELTAS Africa Initiative is an independent funding scheme of the African Academy of Sciences (AAS)'s Alliance for Accelerating Excellence in Science in Africa (AESA) and supported by the New Partnership for Africa's Development Planning and Coordinating Agency (NEPAD Agency) with funding from the Wellcome Trust (UK) and the UK government. The statements made and views expressed are solely the responsibility of the Fellow.

Acknowledgements

All research staff and respondents, management and heads of relevant departments of the University College Hospital, Ibadan are appreciated.

Competing interests

For peer review only - http://bmjopen.bmj.com/site/about/guidelines.xhtml

For peer review only

The authors declare that they have no known competing interests

Authors' contributions

AO conceptualized and developed the protocol for the main study from which the dataset for the current paper was obtained as this formed the research for her doctoral thesis. AO conducted the literature search, and was involved in data collection, analysis and write-up of the current paper. AS and OO were AO's doctoral supervisors. They supervised the entire project and were involved in the write up and revision of the current paper. EA was involved in data collection and write-up of the data for the current paper. TA and GO collated the clinical information of all patients who died in the hospital and generated a list of all-cause mortality for patients managed in the teaching hospital. All authors read and approved the final manuscript.

Data sharing statement

No additional data available

References

1. Habib SH, Saha S. Burden of non-communicable disease: Global overview. Diabetes & Metabolic Syndrome: Clinical Research & Reviews. 2010 2010/01/01/;4(1):41-7.
2. World Health Organization. Rethinking "diseases of affluence"the economic impact of chronic diseases. WHO, 2005.
3. Boutayeb A, Boutayeb S. The burden of non communicable diseases in developing countries. International journal for equity in health. 2005 Jan 14;4(1):2. PubMed PMID: 15651987. Pubmed Central PMCID: PMC546417. Epub 2005/01/18. eng.
4. World Health Organization. Health for the world's adolescents: a second chance in the second decade: summary. World Health Organization, 2014.
5. Alwan A, Maclean DR, Riley L, et al. Monitoring and surveillance of chronic non-communicable diseases: progress and capacity in high-burden countries. Lancet. 2010;376:1861 - 68.
6. Beaglehole R, Bonita R, Horton R, Adams C, Alleyne G, Asaria P, et al. Priority actions for the non-communicable disease crisis. Lancet. 2011 Apr 23;377(9775):1438-47. PubMed PMID: 21474174. Epub 2011/04/09. eng.
7. Jaspers L, Colpani V, Chaker L, van der Lee SJ, Muka T, Imo D, et al. The global impact of non-communicable diseases on households and impoverishment: a systematic review. European Journal of Epidemiology. 2015;30(3):163-88.
8. World Health Organization. Noncommunicable Diseases (NCD) Country Profiles2018. Available from: https://www.who.int/nmh/countries/nga_en.pdf?ua=1.

For peer review only - http://bmjopen.bmj.com/site/about/guidelines.xhtml

9. The Global Cancer Observatory. Nigeria Factsheet. 2020.
10. Adekanle O, Ijarotimi O, Obasi E, Anthony-Nwojo N, Ndububa D. A Southwest Nigerian tertiary hospital 5-year study of the pattern of liver disease admission. NIGERIAN JOURNAL OF GASTROENTEROLOGY AND HEPATOLOGY. 2020 January 1, 2020;12(1):18-23.
11. Rice D. Cost-of-illness studies: fact or fiction? The Lancet. 1994;344(8936):1519-20.
12. Rice DP. Cost of illness studies: what is good about them? Injury Prevention. 2000;6(3):177-9.
13. Segel JE. Cost-of-illness studies—a primer. RTI-UNC Center of Excellence in Health Promotion Economics. 2006:1-39.
14. Rice DP, Kelman S, Miller LS. Estimates of economic costs of alcohol and drug abuse and mental illness, 1985 and 1988. Public health reports. 1991;106(3):280.
15. World Health Organization. WHO guide to identifying the economic consequences of disease and injury. 2009.
16. Gondek K, Lang K, Danchenko N, Shah S, Anderson S, Thompson D. Economic costs of hepatocellular carcinoma in the United States. Journal of Clinical Oncology. 2008;26(15_suppl):6555-.
17. Enstone A, Greaney M, Povsic M, Wyn R, Penrod JR, Yuan Y. The economic burden of small cell lung cancer: a systematic review of the literature. PharmacoEconomics-open. 2018;2(2):125-39.
18. Sheehan DF, Criss SD, Chen Y, Eckel A, Palazzo L, Tramontano AC, et al. Lung cancer costs by treatment strategy and phase of care among patients enrolled in Medicare. Cancer medicine. 2019 Jan;8(1):94-103. PubMed PMID: 30575329. Pubmed Central PMCID: PMC6346221. Epub 2018/12/24. eng.
19. Cipriano LE, Romanus D, Earle CC, Neville BA, Halpern EF, Gazelle GS, et al. Lung cancer treatment costs, including patient responsibility, by disease stage and treatment modality, 1992 to 2003. Value Health. 2011;14(1):41-52. PubMed PMID: 21211485. eng.
20. Nguyen D, Nguyen T, Pho N. Analyzing the treatment cost of liver cirrhosis from healthcare payer's perspective of Vietnam in 2015. Value in Health. 2017;20(9):A633.
21. Rezaei S, Akbari Sari A, Arab M, Majdzadeh R, Mohammad Poorasl A. Economic burden of smoking: a systematic review of direct and indirect costs. Med J Islam Repub Iran. 2016;30:397-. PubMed PMID: 27579287. eng.
22. Muka T, Imo D, Jaspers L, Colpani V, Chaker L, van der Lee SJ, et al. The global impact of non-communicable diseases on healthcare spending and national income: a systematic review. European journal of epidemiology. 2015;30(4):251-77.
23. Mutyambizi C, Pavlova M, Chola L, Hongoro C, Groot W. Cost of diabetes mellitus in Africa: a systematic review of existing literature. Globalization and Health. 2018 2018/01/16;14(1):3.
24. Rice D. Estimating the cost of illness: health economics series No. 6, PHS No. 947–6. Washington, DC: US Government Printing Office. 1966.
25. Chisholm D, Stanciole AE, Tan Torres Edejer T, Evans DB. Economic impact of disease and injury: counting what matters. BMJ. 2010;340.
26. Jedy-Agba E, Curado MP, Ogunbiyi O, Oga E, Fabowale T, Igbinoba F, et al. Cancer Incidence in Nigeria: A Report from Population-based Cancer Registries. Cancer epidemiology. 2012 05/22;36(5):e271-e8. PubMed PMID: PMC3438369.
27. Ladep NG, Taylor-Robinson SD. Management of liver disease in Nigeria. Clinical medicine (London, England). 2007 Oct;7(5):439-41. PubMed PMID: 17990704. Pubmed Central PMCID: PMC4953034. Epub 2007/11/10. eng.
28. CBN. Central Bank of Nigeria: Data and Statistics - Inflation rates n.d. [cited 2017 26 July]. Available from: https://www.cbn.gov.ng/rates/inflrates.asp.
29. CBN. Central Bank of Nigeria: Exchange rate archives: Central Bank of Nigeria; n. d. [cited 2017 6 March, 2017]. Available from: http://www.cbn.gov.ng/rates/ExchangeArchives.asp.

For peer review only - http://bmjopen.bmj.com/site/about/guidelines.xhtml

30. Ezemba N, Ekpe EE, Eze JC. Challenges of lung cancer management in a developing country. Nigerian journal of medicine: journal of the National Association of Resident Doctors of Nigeria. 2012;21(2):214-7.
31. Adewole OO, Anteyi EA, Dosunmu AE, Ajuwon ZA, Erhabor GE, Betiku YL. P1-009: A clinico pathologic features and Management of Lung cancer in a Nigeria. Journal of Thoracic Oncology. 2007;2(8):S553-S4.
32. Nwokediuko S, Osuala P, Uduma U, Alaneme A, Onwuka C, Mesigo C. Pattern of liver disease admissions in a Nigerian tertiary hospital. Nigerian Journal of Clinical Practice. 2013 July 1, 2013;16(3):339-42.
33. Igwegbe AO, Eleje GU, Ugboaja JO, Ofiaeli RO. Improving maternal mortality at a university teaching hospital in Nnewi, Nigeria. International Journal of Gynecology & Obstetrics. 2012 2012/03/01/;116(3):197-200.
34. Ezeome E. Delays in presentation and treatment of breast cancer in Enugu, Nigeria. Nigerian journal of clinical practice. 2010;13(3).
35. Akande TM, Owayemi J. Health-seeking behaviour in Anyigba, North-Central Nigeria. Res J Med Sci. 2009;3:47-51.
36. Almuammar A, Dryden C, Burr J. Factors associated with late presentation of cancer: a limited literature review. Journal of Radiotherapy in Practice. 2010;9(2):117.
37. Idowu OE, Apemiye RA. Delay in presentation and diagnosis of adult primary intracranial neoplasms in a tropical teaching hospital: A pilot study. International Journal of Surgery. 2009 2009/01/01/;7(4):396-8.
38. Edis EC, Karlıkaya C. The cost of lung cancer in Turkey. Tuberkuloz ve Toraks Dergisi. 2007;55(1):51-8.
39. rates.org. E. [20 June, 2020]. Available from: https://www.exchange-rates.org/Rate/USD/VND/12-31-2015.
40. Nwanne C. New minimum wage of N30, 000 takes effect. The Guardian. 2019.

For peer review only

For peer review only - http://bmjopen.bmj.com/site/about/guidelines.xhtml

Table 1: Average direct medical and transportation costs for managing patients who died from selected NCDs in 2020

Duration of treatment (Days)	Average No. of hospital visit (appointments)	Average No. of days on admission	Average direct medical costs per person	Average direct non-medical costs (transportation)	Average direct costs per person
**A**	**B**	**C**	**D**	**E**	**F = D + E**
Lung Ca<br>49.2	1.6	26.7	NGN 484, 445.46<br>USD 1,343.82	NGN 25,707.16<br>USD 71.31	NGN 510,152.62<br>USD 1,415.13
Liver Ca*<br>16.6	1	16.6	NGN 300,764.15<br>USD 834.30	NGN 8,186.12<br>USD 22.71	NGN 308,950.27<br>USD 857.00
Liver cirrhosis<br>21.7	1	13.9	NGN 235,131.27<br>USD 652.24	NGN 2,990.55<br>USD 8.30	NGN 238,121.83<br>USD 660.53

*Duration on treatment (management) **for liver** ca was the same as days on admission for all patients whose records were found because they were all admitted on the day of presentation and stayed on admission until demise.

For peer review only

Appendix 1: Average costs (2020 prices) per patient for a month of terminal care for patients with lung cancer, liver cancer and liver cirrhosis

	Admission history (average)			Average costs for patients										
	Average times of admission	Average hospital visits made	Average days on Admission *	Registration	Consultation	Service Charge	Drugs*	Consumables	Admission	Investigations	Bed side Procedures**	Average Direct Medical	Average Direct Non-medical (transport)	Average Direct medical and non-medical
**Lung Ca (n = 5)**														
2020 naira	1.2	1.6	27.4	1959.83	4442.28	1306.55	13128.24	17798.16	39486.94	354061.35	52262.12	484445.46	25707.16[a]	510152.62
2020 dollar				5.44	12.32	3.62	36.42	49.37	109.53	982.14	144.97	1343.82	71.31	1415.13
**Liver Ca (n = 9)**														
2020 naira	1	1	17.1	1959.83	2467.93	725.86	14404.02	36853.50	24840.48	193381.46	26131.06	300764.15	8186.12[b]	308950.27
2020 dollar				5.44	6.85	2.01	39.96	102.23	68.91	536.43	72.49	834.30	22.71	857.00
**Liver cirrhosis (n = 8)**														
2020 naira	1	1.25	14.6	1959.83	3084.92	907.33	24231.84	18282.67	21231.49	139302.14	26131.06	235131.27	2990.55[c]	238121.82
2020 dollar				5.44	8.56	2.52	67.22	50.71	58.89	386.41	72.49	652.24	8.30	660.53

* Average number of days in total spent in hospital across all of a patient's admissions

a= 4 patients came from other states b = 3 patients came from another state, c =1 patient came from another state

*Drugs listed were mainly supportive for most of the patients

**Examples of bedside procedures - CTTD: Closed tube thoracostomy drainage, liver biopsy etc.

For peer review only

STROBE Statement—checklist of items that should be included in reports of observational studies

	Item No	Recommendation
**Title and abstract**	1	(*a*) Indicate the study's design with a commonly used term in the title or the abstract
		(*b*) Provide in the abstract an informative and balanced summary of what was done and what was found
		Author comment: Abstract included in line with journal specifications Page 3 of 24
**Introduction**		
Background/rationale	2	Explain the scientific background and rationale for the investigation being reported
		Author comment: Information in Background of paper – Pages 5 and 6 of 24
Objectives	3	State specific objectives, including any prespecified hypotheses
		Author comment: Information is in the concluding paragraph of the introduction, just before the results.- Page 6 of 24
**Methods**		
Study design	4	Present key elements of study design early in the paper
		Author comment: Information in opening paragraph of the methods –Page 7 of 24
Setting	5	Describe the setting, locations, and relevant dates, including periods of recruitment, exposure, follow-up, and data collection
		Author comment: In methods – paragraphs 2 and 3 – Page 7 of 24
Participants	6	(*a*) *Cohort study*—Give the eligibility criteria, and the sources and methods of selection of participants. Describe methods of follow-up<br>*Case-control study*—Give the eligibility criteria, and the sources and methods of case ascertainment and control selection. Give the rationale for the choice of cases and controls<br>*Cross-sectional study*—Give the eligibility criteria, and the sources and methods of selection of participants<br>Author comment: Information in paragraph 3 of methods Page 7 of 24
		(*b*) *Cohort study*—For matched studies, give matching criteria and number of exposed and unexposed<br>*Case-control study*—For matched studies, give matching criteria and the number of controls per case
Variables	7	Clearly define all outcomes, exposures, predictors, potential confounders, and effect modifiers. Give diagnostic criteria, if applicable<br>Author comment: Paragraphs 3, 4 and 5 of the methods section on pages 7 to 8 of 24
Data sources/ measurement	8*	For each variable of interest, give sources of data and details of methods of assessment (measurement). Describe comparability of assessment methods if there is more than one group<br>Author comment Paragraphs 3, 4 and 5 of the methods section on pages 7 to 8 of 24
Bias	9	Describe any efforts to address potential sources of bias<br>Author comment: Medical and transportation costs were obtained from at least two sources. Information presented in Pages 7 and 8 of 24
Study size	10	Explain how the study size was arrived at<br>Author comment: Primary data was not collected and all available records found were analysed.
Quantitative variables	11	Explain how quantitative variables were handled in the analyses. If applicable, describe which groupings were chosen and why

For peer review only - http://bmjopen.bmj.com/site/about/guidelines.xhtml

		Author comment: Information on variables is provided in the section just preceding the ethical approval section Pages 7 to 9 of 24
Statistical methods	12	(*a*) Describe all statistical methods, including those used to control for confounding
		(*b*) Describe any methods used to examine subgroups and interactions
		(*c*) Explain how missing data were addressed
		(*d*) *Cohort study*—If applicable, explain how loss to follow-up was addressed<br>*Case-control study*—If applicable, explain how matching of cases and controls was addressed<br>*Cross-sectional study*—If applicable, describe analytical methods taking account of sampling strategy
		(*e*) Describe any sensitivity analyses
		Author comment: Information presented in the methods section – the sub-section just preceding the results Pages 8 and 9 of 24

Continued on next page

For peer review only

**Results**		
Participants	13*	(a) Report numbers of individuals at each stage of study—eg numbers potentially eligible, examined for eligibility, confirmed eligible, included in the study, completing follow-up, and analysed - Methods section Page 7 of 24 second paragraph
		(b) Give reasons for non-participation at each stage
		(c) Consider use of a flow diagram
Descriptive data	14*	(a) Give characteristics of study participants (eg demographic, clinical, social) and information on exposures and potential confounders
		(b) Indicate number of participants with missing data for each variable of interest
		(c) *Cohort study*—Summarise follow-up time (eg, average and total amount) Page 10. Opening paragraph of the results section: Page 10 of 24
Outcome data	15*	*Cohort study*—Report numbers of outcome events or summary measures over time
		Case-control study—Report numbers in each exposure category, or summary measures of exposure
		Cross-sectional study—Report numbers of outcome events or summary measures
Main results	16	(*a*) Give unadjusted estimates and, if applicable, confounder-adjusted estimates and their precision (eg, 95% confidence interval). Make clear which confounders were adjusted for and why they were included
		(*b*) Report category boundaries when continuous variables were categorized
		(*c*) If relevant, consider translating estimates of relative risk into absolute risk for a meaningful time period Not applicable
Other analyses	17	Report other analyses done—eg analyses of subgroups and interactions, and sensitivity analyses
		Author comment: Information presented in the results section – Pages 10 to 11 of 24
**Discussion**		**Author comment: Information presented in the results section – Pages 11 to 13 of 24**
Key results	18	Summarise key results with reference to study objectives
Limitations	19	Discuss limitations of the study, taking into account sources of potential bias or imprecision. Discuss both direction and magnitude of any potential bias Author comment: Information presented after the concluding sub-section –Pages 13 and 14 of 24
Interpretation	20	Give a cautious overall interpretation of results considering objectives, limitations, multiplicity of analyses, results from similar studies, and other relevant evidence Author comment: Information provided in the discussion section - Pages 11 to 13 of 24
Generalisability	21	Discuss the generalisability (external validity) of the study results
		Author comment: Information presented in the conclusion section Page 13 of 24
**Other information**		
Funding	22	Give the source of funding and the role of the funders for the present study and, if applicable, for the original study on which the present article is based Author comment: Information presented after the limitations Page 14 of 24

*Give information separately for cases and controls in case-control studies and, if applicable, for exposed and unexposed groups in cohort and cross-sectional studies.

Note: An Explanation and Elaboration article discusses each checklist item and gives methodological background and published examples of transparent reporting. The STROBE checklist is best used in conjunction with this article (freely available on the Web sites of PLoS Medicine at http://www.plosmedicine.org/, Annals of Internal Medicine at

For peer review only - http://bmjopen.bmj.com/site/about/guidelines.xhtml

http://www.annals.org/, and Epidemiology at http://www.epidem.com/). Information on the STROBE Initiative is available at www.strobe-statement.org.

For peer review only
